# Putative Cooperative ATP–DnaA Binding to Double-Stranded DnaA Box and Single-Stranded DnaA-Trio Motif upon *Helicobacter pylori* Replication Initiation Complex Assembly

**DOI:** 10.3390/ijms22126643

**Published:** 2021-06-21

**Authors:** Pawel Jaworski, Dorota Zyla-Uklejewicz, Malgorzata Nowaczyk-Cieszewska, Rafal Donczew, Thorsten Mielke, Christoph Weigel, Anna Zawilak-Pawlik

**Affiliations:** 1 Department of Microbiology, Hirszfeld Institute of Immunology and Experimental Therapy, Polish Academy of Sciences, Weigla 12, 53-114 Wrocław, Poland; paweljaworski1988@gmail.com (P.J.); dorota.uklejewicz@gmail.com (D.Z.-U.); mnowaczykm@gmail.com (M.N.-C.); rafal.donczew@gmail.com (R.D.); 2 Microscopy and Cryo-Electron Microscopy Service Group, Max Planck Institute for Molecular Genetics, Ihnestrasse 63-73, 14195 Berlin, Germany; mielke@molgen.mpg.de; 3 Institute of Biotechnology, Faculty III, Technische Universität Berlin (TUB), Straße des 17. Juni 135, 10623 Berlin, Germany; christoph.weigel.berlin@gmail.com

**Keywords:** *oriC*, orisome, DnaA box, initiation of chromosome replication

## Abstract

*oriC* is a region of the bacterial chromosome at which the initiator protein DnaA interacts with specific sequences, leading to DNA unwinding and the initiation of chromosome replication. The general architecture of *oriC*s is universal; however, the structure of *oriC* and the mode of orisome assembly differ in distantly related bacteria. In this work, we characterized *oriC* of *Helicobacter pylori*, which consists of two DnaA box clusters and a DNA unwinding element (DUE); the latter can be subdivided into a GC-rich region, a DnaA-trio and an AT-rich region. We show that the DnaA-trio submodule is crucial for DNA unwinding, possibly because it enables proper DnaA oligomerization on ssDNA. However, we also observed the reverse effect: DNA unwinding, enabling subsequent DnaA–ssDNA oligomer formation—stabilized DnaA binding to box ts1. This suggests the interplay between DnaA binding to ssDNA and dsDNA upon DNA unwinding. Further investigation of the ts1 DnaA box revealed that this box, together with the newly identified c-ATP DnaA box in *oriC1*, constitute a new class of ATP–DnaA boxes. Indeed, in vitro ATP–DnaA unwinds *H. pylori oriC* more efficiently than ADP–DnaA. Our results expand the understanding of *H. pylori* orisome formation, indicating another regulatory pathway of *H. pylori* orisome assembly.

## 1. Introduction

Chromosomal DNA replication is an essential cellular process that is tightly regulated at the initiation step to ensure that it occurs once and only once per cell cycle [1,2,3]. The fundamental mechanism of the initiation of DNA replication is common to the majority of bacteria. Replication begins with the binding of the initiator protein DnaA to a specific chromosomal region termed *oriC* [4]. DnaA oligomerization leads to the assembly of a highly organized nucleoprotein complex that remodels the *oriC* structure and triggers duplex destabilization within an adjacent, helically-unstable AT-rich region termed the DNA unwinding element (DUE) [5,6,7]. The emergent replication bubble is subsequently stabilized by SSB and DnaA interactions and serves as the recruitment site for the enzymes responsible for strand separation and DNA synthesis [8,9,10,11].

The DnaA protein consists of four structural domains responsible for different but mutually dependent functions (see [3,12,13] and references therein). N-terminal domain I is important for DnaA dimerization and interactions with other proteins. Domain II is a flexible linker connecting domain I with the C-terminal domains III and IV. Domain III, which belongs to a family of ATPases associated with diverse cellular activities (AAA+), binds ATP/ADP and is responsible for ATP-dependent DnaA oligomerization. Domain III is also responsible for interactions with single-stranded DNA in a DUE via initiator-specific motif (ISM; ISM is also known as the H-motif) and the B-motif; the ISM and B motifs are also known as the H/B motif [14,15,16]. Domain IV is responsible for the binding of the double-stranded (ds) DnaA box.

The most comprehensive descriptions of the bacterial chromosome replication process and orisome structure come from studies on *Escherichia coli* [2]. The *E. coli* origin of replication is continuous and encompasses approximately 250 bps [17]. It comprises a DUE and a cluster of DnaA binding sites (DnaA boxes) [2,12]; the regulatory sequences have been described elsewhere [18]. The cluster of DnaA boxes is subdivided into two arrays (left and right) of oppositely oriented DnaA boxes [10,19], which are used as a scaffold for DnaA oligomerization (see below). Each array contains a single 9-mer high-affinity R-type DnaA box (R1 and R4, respectively) and several low-affinity boxes (R5M, τ1-2, I1-3, c1-c3); DnaA box R2, which displays moderate affinity, is located between the arrays. The R-type DnaA boxes (consensus 5′-TTATMCACA-3′) are bound by DnaA complexed with ATP or ADP (ATP–DnaA or ADP–DnaA) and serve as anchors for subsequent DnaA-binding low-affinity boxes [20,21]. The low-affinity sites are bound by ATP–DnaA, which is important for the synchronization and/or regulation of chromosome replication [22,23,24]. The low-affinity ATP–DnaA boxes differ by 3–6 bases from the R-type DnaA box [19,25]. Comparison of the sequences of the low-affinity sites indicates that some of these boxes share the core sequence (5′-TGATCC-3′, 2-7 nt of a 9-mer) [24,25], whereas the remaining low-affinity DnaA boxes resemble highly degenerate R binding sites. The binding of ATP to DnaA alters the protein’s structure, especially that of domains III and IV, increasing the affinity of DnaA for DNA and causing DnaA to oligomerize into a filament, which in turn enables separation of the DNA strands [26,27,28,29,30,31]. The DnaA filament is highly organized into inward-oriented DnaA left- and right-subcomplexes of different functions [32,33]. The left DnaA pentamer, especially the protomers bound to DnaA boxes R1 and R5M, is important for DNA unwinding and ssDUE binding [34]. The *E. coli* DUE region contains six 6-mer ATP–DnaA boxes (5′-AGATCT-3′) that are bound exclusively in the single-stranded form [35] and two T-rich sequences (5′-TTATT-3′ and 5′-TTGT-3′) that are crucial for maintaining the open complex [14]. The right DnaA filament, formed by the binding of ATP–DnaA to DnaA boxes R4, C1-C3, and I3, also interacts with ssDNA, but it is primarily engaged in DnaB loading [10]. After completing the *E. coli* replisome machinery [9], DnaA is inactivated by ATP hydrolysis stimulated by DnaA–DnaN–Hda tripartite interactions, preventing premature reinitiation [36].

**Figure 1:**
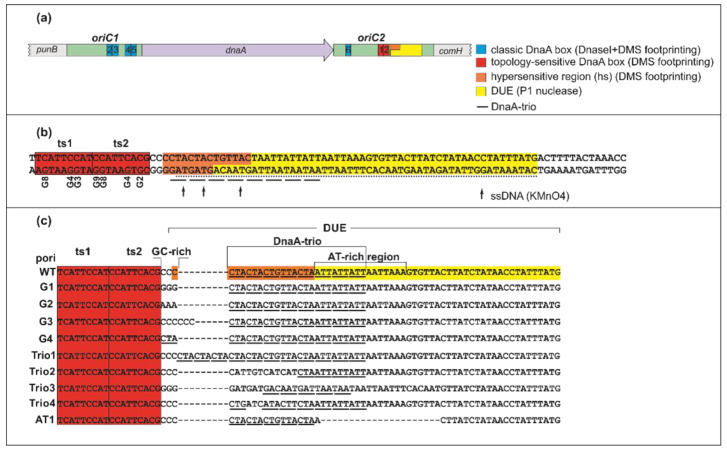
*H. pylori* bipartite origin. (**a**) Schematic presentation of *H. pylori* bipartite *oriC*. Important origin modules are marked. See also the Introduction and [37,40] for details. (**b**) The sequence of the ts boxes-DUE region. Potassium permanganate (KMnO_4_) sensitive sites are marked (see also Figure S1). The guanine residues (G) in ts boxes modified by dimethyl sulfate (DMS) in footprinting analyses are given below the sequence (5′-3′ numbering of the bottom strand residues). (**c**) Schematic representation of the modifications introduced into the pori plasmids. The DNA unwinding element (DUE) and hs regions (highlighted yellow and orange, respectively) are only marked for poriWT plasmids; they were determined experimentally in this work for other mutated plasmids.

The *E. coli* model of chromosome replication initiation was proposed for a continuous *oriC* structure; the structure of the orisome formed on a bipartite *oriC* might differ. Both *Helicobacter pylori* (Figure 1A) and *Bacillus subtilis oriC*s are distinguished by a bipartite origin arrangement with two DnaA box clusters (*oriC* subregions 1 and 2) separated by the *dnaA* gene [37,38]. DnaA binds to both subregions, and due to DnaA–protein interactions between subcomplexes, loops out the *dnaA* gene [37,38]. The *oriC1* (*incAB*) of *B. subtilis* encompasses 620 bps and contains 13 DnaA boxes, whereas *oriC2* (*incC*) is 189 bp in length and consists of 7 DnaA binding sites (DnaA boxes 1-7) and the DUE [11,38,39]. Not all DnaA boxes are required for the initiation of *B. subtilis* chromosome replication; however, *oriC2* DnaA box 6 is essential but not sufficient for the origin replication in vivo [39]. The unwound region of *B. subtilis oriC* consists of six DnaA-trio repeats located at the beginning of the DUE [11], followed by an AT-rich region. DnaA-trio (stringent consensus sequence 3′-GAT-5′) is a sequence bound as single-stranded DNA by a DnaA filament upon DUE unwinding. The DUE and the R1*_E. coli_*-type DnaA box (*B. subtilis* DnaA box 7) are separated by a GC-rich motif (5′-GGCC-3′) whose function remains unknown; please note that by the R1*_E. coli_*-type DnaA box, we assume the DUE proximal DnaA box as the box R1 in *E. coli oriC*, regardless of the sequence identity. The *H. pylori oriC1* region (152 bps) contains four classic (c) topology-insensitive DnaA boxes c2-c4 with the conserved 5′-TYATTCACN-3′ consensus sequence (Figure 1A) [40]. *OriC2* (228 bps) contains three DnaA boxes—one classic (c6) and two topology-sensitive (ts) binding sites (ts1, ts2)—and the DUE module located downstream of the ts boxes (Figure 1A,B) [40]. DnaA box ts2 directly precedes the DUE module; thus, it constitutes an R1*_E. coli_*-type DnaA box. The DnaA box ts1 is a low-affinity binding site that requires interactions with DnaA bound to DnaA box ts2 [40]. The *H. pylori* DUE module is composed of a DnaA-trio submodule [11]. The DnaA-trio submodule is separated from the DnaA box ts2 by a GC-rich sequence and is followed by an AT-rich region [37] that begins with three 5′-ATT-3′ triplets, which may constitute an imperfect DnaA-trio. The *oriC1* region is nonessential for DUE opening in vitro [37], but it interacts with *oriC2* via DnaA bound to box c6, resulting in DNA loop formation and remodeling of DUE-proximal *oriC2*; this can be observed as hypersensitivity of the DNA to DMS (hs region) [40]. However, the reason for this hypersensitivity remains unclear.

The mechanism of DnaA binding to dsDNA and the rules of oligomer formation are relatively well known. In contrast, the mechanisms of DNA unwinding and subsequent DnaA–ssDNA interactions, especially in the context of the entire initiation complex, are still not fully understood. Two basic models describe DNA unwinding and ssDNA binding by DnaA. The “ss-DUE recruitment model,” which is based on studies of the *E. coli* initiation complex [33,34], proposes that upon DNA unwinding, the same DnaA filament that binds dsDNA via domain IV opens the DNA and subsequently simultaneously binds ssDNA via multiple H/B motifs arranged by domains III of DnaA. Indeed, it has recently been shown that *E. coli* DnaA molecules bound to boxes R1 and R5M are crucial for interaction with ssDNA in an H/B motif-dependent manner [34]. An alternative “DnaA continuous filamentation” model derived from studies of *A. aeolicus* DnaA proposes that DnaA forms two types of DnaA filaments, dsDNA–DnaA and ssDNA–DnaA, that differ in DnaA structure [5,6,15,31]. The dsDNA–DnaA filament opens DNA and promotes the formation of a second filament on ssDNA; however, the DnaA molecules bound to ssDNA and dsDNA, respectively, do not interact. Therefore, the general difference between the two models is the putative capability of the DnaA molecule to bind ssDNA and dsDNA simultaneously. A third “alternative” model proposes that there are two DnaA filaments, dsDNA–DnaA and ssDNA–DnaA, that interact via filament-engaged DnaA molecules [34].

This study aimed to characterize the bipartite *oriC* of *H. pylori* further, focusing on the DUE and the DUE-proximal region and their roles in DNA unwinding. The influences of the GC-rich sequence, the DnaA-trio, and the AT-rich region on DNA unwinding and DnaA binding to the DUE-proximal DnaA boxes were confirmed by P1 nuclease and dimethyl sulfate (DMS) footprinting. We found that the DnaA-trio region, and presumably, oligomer formation on ssDNA are crucial for stabilizing DnaA binding to the dsDnaA box ts1. The DUE unwinding and binding of ts1 are dependent on ATP. Interestingly, there are two ATP–DnaA boxes within the *H. pylori oriC*, one at each subregion: ts1 at *oriC2* and c-ATP at *oriC1.* These two DnaA boxes share a sequence (5′-TYATTCCWT-3′) typical of ATP–DnaA boxes [12,41]. We propose that ts1 might play a role in ssDNA stabilization and that boxes ts1 and c-ATP might both be important for ATP-dependent regulation of *H. pylori* chromosome replication.

## 2. Results

### 2.1. DnaA Unwinds H. pylori OriC at the First DnaA-Trio Motif

*OriC* of *H. pylori* and most of the studied Epsilonbacterota contain DnaA-trio motifs in the region adjacent to the R1*_E. coli_*-type box [42]. The DUE sequence of *H. pylori oriC,* previously identified using the P1 nuclease test [37], encompasses approximately 50 nt and begins at the third repeat of the DnaA-trio, 10 nt downstream of box ts2 (Figure 1A,B).

It has recently been shown in *B. subtilis* that a DnaA filament is built on the first ssDNA DnaA-trio motif immediately downstream of the DnaA boxes [11]. Moreover, the DnaA-trio motifs proximal to *B. subtilis* DnaA box 7 (corresponding to DnaA box R1 in *E. coli*) are more important than the distant motifs for the initiation of chromosome replication and DnaA filament formation. This discrepancy between *B. subtilis* and *H. pylori* DNA unwinding sites encouraged us to reexamine ssDNA formation using potassium permanganate (KMnO_4_) chemical modification, which permits monitoring of DNA helix status (ds- or ssDNA) ([43,44,45], Supplementary Materials and Methods and Figure S1). KMnO_4_ preferentially modifies pyrimidine bases, specifically thymine residues, located within single-stranded DNA. Using supercoiled pori1ori2 plasmid [37] (herein referred to as poriWT, Table S1), we identified modified thymines located in the first, second, and fourth DnaA-trio repeats (Figure 1B and Figure S1). The base specificity of KMnO_4_ oxidation precludes identification of ssDNA formation in the third DnaA-trio motif in *H. pylori* (3′-GAC-5′), which lacks a thymine residue (please note that we monitor DnaA binding to the lower strand of the DnaA-trio, which is presented by primer extension as a sequence of the complementary strand; thus, in the present work, the perfect 3′-GAT-5′ DnaA-trio is regarded as a complementary 5′-CTA-3′ sequence). Notably, the ssDNA sequence detected by KMnO_4_ footprinting overlaps with the hs region. The overlap of the hs site and the ssDNA detected by KMnO_4_ suggests that the hypersensitivity of DNA observed in DMS footprinting resulted from DNA unwinding or severe distortion of its double helix. It has been shown that the formation of ssDNA also changes the chemical environment, increasing the sensitivity of purines to DMS treatment, in this case N7 of guanine [46]. In conclusion, upon DnaA–*oriC* interaction, DnaA unwinds DNA starting 3 nt downstream of DnaA box ts2; the DUE extends for approximately 60 nt.

### 2.2. The DnaA-Trio Influences DUE Unwinding and DnaA Binding to dsDNA DnaA Box ts1

The DnaA-trio motif, together with DnaA box 6 of the *B. subtilis oriC2* region, is important for the assembly of a DnaA protein filament on ssDNA [11,39]. Thus, the DnaA-trio is presumably responsible for stabilizing the emergent replication bubble in *B. subtilis* and multiple unrelated bacterial species containing the DnaA-trio, including *H. pylori*. However, most of the reported in vitro experiments involving the DnaA-trio have focused on monitoring DnaA oligomerization on short, linear DNA oligonucleotides [11,39]. Thus, the influence of the DnaA-trio on in vitro DNA unwinding in the context of the entire *oriC* has not been characterized in detail. In addition, the role of the GC-rich sequence that often accompanies the DnaA-trio has not been studied. Thus, we decided to analyze the effects of mutations in the *H. pylori* DUE submodules (the GC-rich sequence, the DnaA-trio, and the AT-rich region) (Figure 1C, Table S1) on DNA unwinding and DnaA–DNA interactions in vitro. The mutations were designed such that potential differences between wild-type and mutated DUE would arise exclusively from the intended nucleotide exchange within the analyzed DUE submodule rather than from an overall abrogation of the DUE function (Figure S2). Mutations of the GC-rich sequence (poriG1-4) included the exchange of C nucleotides for G or A (poriG1 and poriG2, respectively), elongation of the GC-rich sequence, increasing the distance between the GC-rich sequence and DnaA box ts2 (poriG3), and exchange of the GC-rich sequence for a DnaA-trio (poriG4) (Figure 1C). The mutations at the DnaA-trio introduced additional DnaA-trio repeats (poriTrio1) or modified the sequence of the DnaA-trio submodule (poriTrio2-4). In poriAT1, the AT-rich sequence was deleted.

**Figure 2:**
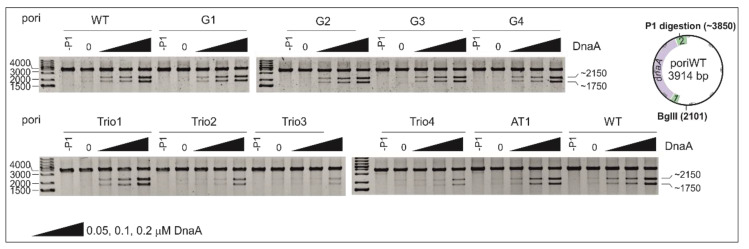
DNA unwinding of pori plasmids with modified DnaA-trio motifs, AT-rich, or GC-rich regions. In the nuclease assay, the plasmids were incubated with the indicated amounts of DnaA, digested by P1 nuclease and digested by BglII (P1/BglII). Each of the experimental sets (upper and lower panels) included the poriWT plasmid as a reference. The DNA fragments were resolved on 1% agarose gels and stained with ethidium bromide. The sizes of the GeneRuler 1 kb Plus DNA ladder bands and the expected DNA restriction fragments (in base pairs) are indicated to the left and right of the gel images, respectively. -P1, sample without DnaA and without P1 nuclease.

A P1 nuclease assay was employed to determine the effect of the introduced mutations on the efficiency of DUE unwinding (Figure 2).

Unlike *E. coli* DnaA, which requires the assistance of HU protein to unwind DUE [47], *H. pylori* DnaA unwinds *oriC* alone [37,40], and HU does not significantly affect DnaA unwinding activity (Figure S3). Thus, in our analyses, we used the most basic components of the P1 unwinding reaction. The wild-type and mutated plasmids were incubated with increasing concentrations of DnaA and treated with P1 nuclease, which digests single-stranded DNA. The P1-treated plasmids were further digested by BglII (P1/BglII) and resolved on agarose gels. The results of the P1/BglII analysis indicate that neither any modifications introduced within the GC-rich sequence (poriG1-4) nor deletion of 21 bps in the AT-rich module (poriAT1) significantly influenced the ability of DnaA to unwind the DUE region in comparison to poriWT (Figure 2). The introduction of three additional DnaA-trio repeats between the GC-rich sequence and the DUE (poriTrio1) did not affect the plasmid’s P1 susceptibility compared to the poriWT plasmid. Disruption of the motif or reversal of polarity of the DnaA-trio submodule (poriTrio2 and poriTrio3, respectively) inhibited the formation of ssDNA, indicating the relevance of the DnaA-trio motifs to DUE unwinding and possibly to assembly of the DnaA oligomer on the separated ssDNA strands. For the poriTrio4 plasmid containing a randomized DnaA-trio motif in which the variable nucleotides at the 1st and 3rd positions of the 5′-CTA-3′ motif were changed but the strictly conserved thymine residue was not modified, digestion of the DNA by P1 nuclease did not occur. This corroborates the hypothesis that although the residues at the 1st and 3rd positions are less conserved than the residue at the 2nd position, they are also important for DnaA-trio binding, and as a consequence, for DNA unwinding.

To characterize the effects of the mutations on DUE unwinding and DnaA-DNA interactions at the nucleotide level, we used P1 digestion of DnaA-plasmid complexes followed by primer extension (P1/PE analysis) and DMS footprinting. P1 nuclease allows mapping of ssDNA; however, as shown by Speck and Messer, ssDNA might be protected from digestion by P1 nuclease by the DnaA filament assembled on the unwound strand [35]. Therefore, P1/PE analysis may indirectly determine the length and the stability of the DnaA filament. DMS footprinting was used to complement the results of P1/PE. DMS allows one to determine the presence of ssDNA and maps the borders of the unwound or distorted DNA, but it simultaneously allows monitoring of DnaA binding to dsDNA. This method is based on the predominant methylation of guanine (and to a lesser degree of adenine and cytosine) [48], increasing the susceptibility of the proximal phosphodiester bonds in the DNA backbone to piperidine cleavage. Interaction between DNA and protein or ssDNA formation alters the DMS modification pattern, and in consequence, leads to a decrease or an increase in nucleic acid fragmentation. The efficiency of DNA cleavage at the binding site is subsequently detected by a primer extension reaction. Thus, P1/PE and DMS footprinting are complementary methods that allow precise monitoring of DNA binding and unwinding by DnaA, and indirectly, measuring of DnaA oligomer length or stability. It should be noted that combined assay of P1/PE and DMS footprinting is one of the few methods of the analysis of DnaA interactions with the full-length supercoiled *oriC* (*oriC1-dnaA-oriC2*) in which DnaA simultaneously oligomerizes on dsDNA and ssDNA and assembles into a complex nucleoprotein structure.

**Figure 3:**
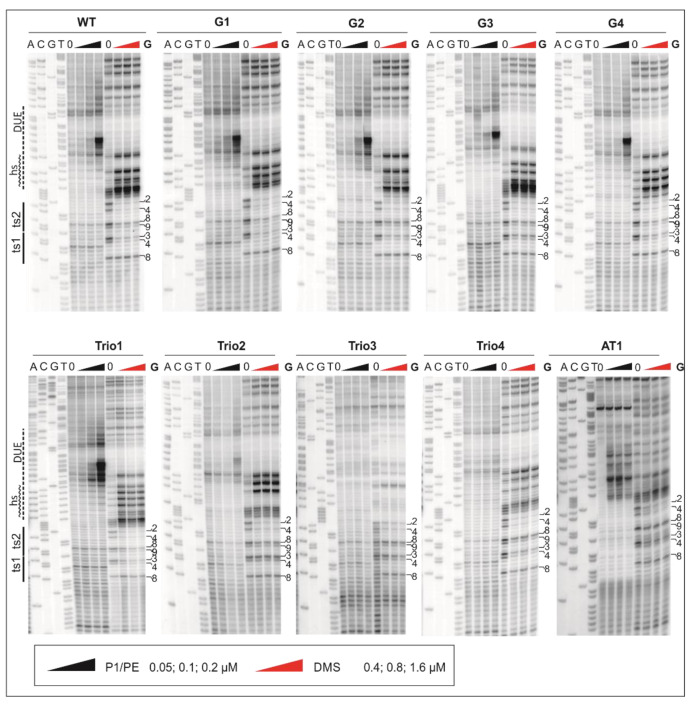
Analysis of DnaA binding to the 5′ DUE border and DUE-proximal boxes. After incubation with the indicated amounts of DnaA, plasmid were treated with P1 nuclease or DMS and used as templates in PE reactions. The DUE and ts boxes are marked to the left of the gels; DMS-modified guanine (G) residues in ts1 and ts2 DnaA boxes are labeled to the right of the gels.

The plasmids encoding wild-type and mutated DUE were incubated with DnaA and treated with P1 nuclease or DMS. Subsequently, the P1-digested products and the DMS-treated plasmids were analyzed in PE reactions using the E1 primer complementary to the upstream region of DUE to simultaneously monitor the formation of ssDNA and indirectly DnaA filament assembly and measure changes within the ts DnaA boxes and the DUE submodules (Figure 3). Please note that we focused on the 5′ border of the DUE only (ts boxes, GC-rich sequence, and DnaA-trio submodule); the 3′ border, marked in [37], was beyond the scope of this research.

**Figure 4:**
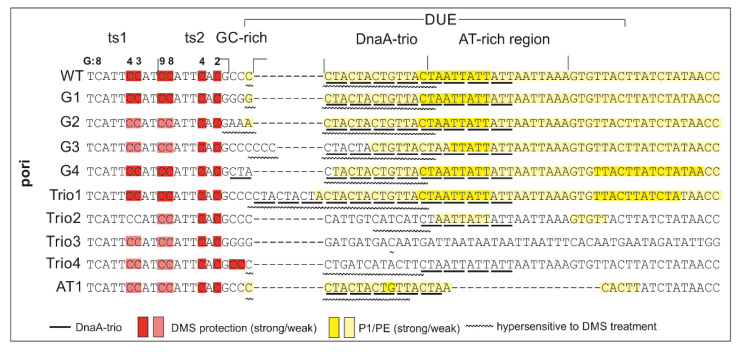
Summary of DnaA-trio analyses in *H. pylori* conducted in this study. The most important features of the analysis are indicated as follows: the red and pink rectangles depict protection of DnaA boxes upon protein binding and decreased interaction in comparison with the poriWT sequence, respectively; the intensity of the yellow rectangles indicates the susceptibility of DNA strands to P1 nuclease digestion and the frequency of the open complex formation; wavy lines indicate sequences that were hypersensitive to DMS methylation upon DnaA binding.

In the poriWT P1/PE analysis, PE products were detected starting from the 3rd residue downstream of DnaA box ts2, and they extended along approximately 24 nt in the 3′ direction from DnaA box ts2, indicating the presence of the 22-nt ssDNA (Figure 4).

**Figure 5:**
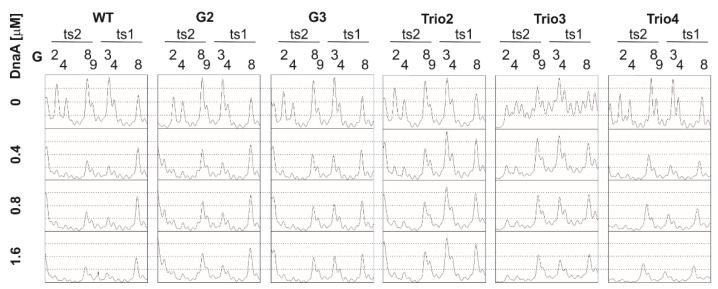
Densitometric analysis of the DMS gels (Figure 3) presenting disturbed DnaA interaction with DnaA boxes at the mutated *oriC2*; WT *oriC2* is shown as a control. The concentrations indicated next to the plots correspond to the analyzed lanes. The protected guanine residues (G) and the protected positions within each DnaA box are indicated above the plots.

Weak PE products were detectable over the first 13 nt, whereas the major PE product was detected at the 3′ border of the DnaA-trio submodule. The observed PE product pattern suggests that the relatively stable DnaA filament extended over 13 nt, encompassing the first four DnaA-trio interactions at the 5′ DnaA-trio submodule, and allowed only limited P1 digestion of the DNA. The interactions of DnaA with the four DnaA-trio repeats at the 3′ region of the submodule was possibly weaker, and P1 digestion was more effective, yielding a strong P1/PE signal. In DMS footprinting, the hs region started approximately at the 3rd C residue downstream of DnaA box ts2 and extended over the next 15 nt, encompassing the first five DnaA-trio repeats. ssDNA might extend as determined in the P1/PE analysis, but the lack of C residues in the AT-rich region (G residues in the bottom strand, which is extended by polymerase) renders the detection of ssDNA impossible. Thus, P1/PE and DMS footprinting results indirectly showed that DnaA binds the four or five ssDNA DnaA-trio motif repeats. The DnaA boxes were protected from DMS methylation as previously shown; i.e., the G3 and G4 of ts1, and G2, G4, G8, and G9 residues of DnaA box ts2 were protected [40] (for G-residue numbers in ts1 and ts2 boxes; see Figure 1B, Figure 3, Figure 4 and Figure 5 and Figure S4). Please note that G8 and G9 residues are visible as single-band or double-band signals [40,49], and double-band protection is more visible than a single-band. At this stage of research, we cannot explain this discrepancy.

Next, the plasmids of the poriG1-G4 series were analyzed. The P1/PE products extended approximately up to 24 nt downstream of the ts2 DnaA box in each plasmid, indicating similar stability and length of the DnaA oligomer as in the poriWT (Figure 3, Figure 4 and Figure 5 and Figure S4). The hs regions in poriG1, poriG3, and poriG4 remained unchanged; however, in poriG2, the hs region was moved by 3 nt towards the ts2 DnaA box, possibly due to deletion of the GC-rich sequence that reduced the stability of dsDNA downstream of the ts2 DnaA box. DnaA boxes in poriG1 and poriG4 were protected as in the poriWT. Interestingly, the DMS footprinting indicated that in the poriG2 and poriG3, the G2 and G4 residues of DnaA box ts2 were bound similarly to that in the poriWT plasmid, but the DnaA interactions with G3/G4 and G8/G9 residues of DnaA boxes ts1 and ts2, respectively, were decreased (Figure 3, Figure 4 and Figure 5 and Figure S4; please compare the intensities of the bands/peaks of the above-mentioned guanine residues of the ts1-ts2 boxes with the band/peak representing the non-interacting G8 residue of the ts1 box). Therefore, we conclude that substitution of the GC-rich sequence by the AT-rich sequence—and in consequence premature DNA unwinding or elongation of the GC-rich tract—and oligomer formation shifting away from the ts2 DnaA box probably affected heretofore uncharacterized cooperation between proteins bound to the DnaA-trio and ts1 and G9/G8 residues of the box ts2.

Next, the plasmids of the poriTrio1-Trio4 series were analyzed. The increased number of DnaA-trio repeats in poriTrio1 resulted in the formation of elongated oligomers assembled along 31 nt encompassing seven DnaA-trio repeats. The major P1/PE products were detected exactly at the same region as in the poriWT, i.e., within four last DnaA-trio repeats, which suggests that the DNA sequence (i.e., the lack of DnaA-trio motif) determines the end of the DnaA oligomer at the 3′ end or downstream of the DnaA-trio submodule. In other words, a DnaA filament is formed on ssDNA using the consecutive DnaA-trio motifs as long as they are present. The interactions of DnaA with the ts boxes of poriTrio1 remained unchanged. However, the major mutations in the DnaA-trio sequence led to marginal unwinding (poriTrio2) or lack of DUE opening (poriTrio3 and poriTrio4), confirming the results obtained by P1/BglII agarose gel analysis. DMS hypersensitive region in poriTrio2 was shifted away from ts boxes compared to the poriWT, and unlike in plasmids undergoing unwinding, the G residues in the GC-rich region were not significantly modified. There was only one G residue in the modified DnaA-trio region in poriTrio3; thus, it was impossible to determine the hs region modification. However, in poriTrio4, there were four consecutive guanine residues at the GC-rich region, which means that when modified, they should be clearly visible, as in the case for poriWT. However, they were barely modified by DMS (please compare the intensity of the bands in the hs region of poriTrio4 and poriWT). Altogether, P1/PE and DMS analyses indicated that without the DnaA-trio submodule, DnaA could not unwind and stabilize ssDNA, possibly because DnaA cannot assemble into the correct oligomer on ssDNA (Figure 3 and Figure 5). Interestingly, in all three plasmids, the DnaA interacted with G2/G4 of DnaA box ts2; however, the interactions with G8/G9 of DnaA box ts2 and G3/G4 of DnaA box ts1 were markedly reduced (Figure 3, Figure 4 and Figure 5 and Figure S3), indicating that DnaA bound to the DnaA-trio submodule stimulated or stabilized the interaction of DnaA with dsDNA box ts1 and the 5′ sequence of the DnaA box ts2.

Deleting the AT-rich submodule (poriAT1) did not change the DNA melting start site (Figure 3, Figure 4 and Figure 5), leading to the conclusion that the DnaA-trio repeats rather than the AT-rich part of the DUE region are important for initial DNA opening. The binding of DnaA to DnaA box ts2 remained unchanged, but the interaction with DnaA box ts1 was weakened, probably due to the reduced size of the DUE or due to the lack of imperfect DnaA-trio repeats (5′-ATT-3′).

In summary, the DnaA-trio motif disruption prevents DNA strand separation, probably due to improper formation or lack of DnaA oligomer formation on ssDNA that stabilizes unwound DNA. Lack of ssDNA oligomer formation (no correct DnaA-trio in poriTrio2-Trio4) or an incorrect unwinding site (disturbed in poriG2) or incorrect positioning of the oligomer in relation to ts1 and ts2 DnaA boxes (disturbed in poriG3) reduces the interaction of DnaA with DnaA box ts1 and the 5′ region of DnaA box ts2, suggesting an interaction between DnaA bound to ts1 and ts2 DnaA boxes and ssDNA.

### 2.3. H. pylori OriC Contains Two ATP-Dependent DnaA Boxes

The inhibited or reduced binding of DnaA box ts1 by DnaA observed for plasmids with mutated DnaA-trio (poriTrio2-4) or AT-rich (poriAT1) and GC-rich (poriG2-G3) submodules, respectively, encouraged us to investigate further the mechanism of DnaA interaction with the low-affinity DnaA box ts1, which requires DnaA box ts2 for efficient binding [40]. The ts1 sequence (5′-TCATTCCAT-3′) differs from classic *H. pylori* DnaA boxes in the presence of a double cytosine in the conserved portion of the DnaA box sequence. Such a sequence is found in ATP–DnaA boxes that are crucial in regulating DNA synthesis initiation in multiple bacterial species (e.g., *E. coli* and *Caulobacter crescentus*) [12,41]. To determine whether *H. pylori* DNA unwinding by DnaA is also ATP-dependent and to determine whether (and which) DnaA boxes are crucial for ATP-dependent DnaA binding to *oriC*, we used P1 nuclease assays and DMS footprinting.

**Figure 6:**
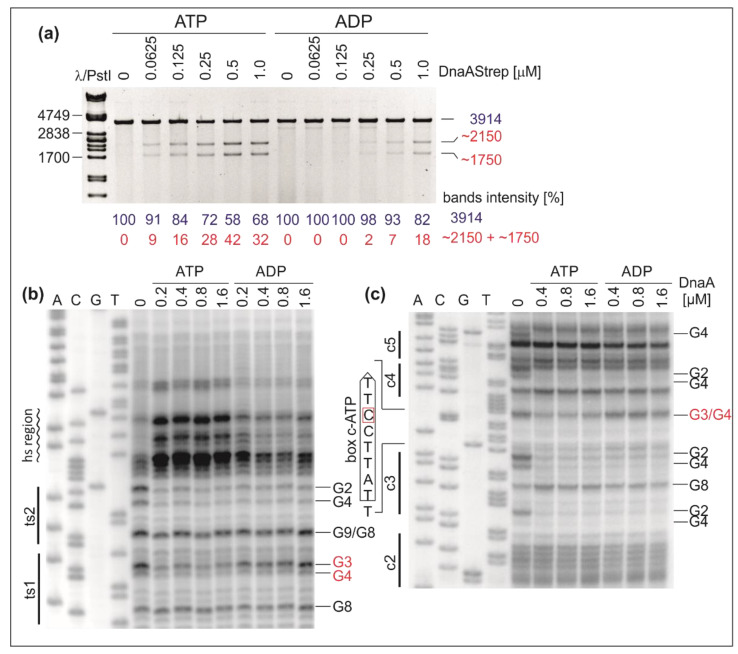
P1 nuclease analysis of DUE unwinding in the presence of ATP/ADP and identification of ATP-dependent DnaA boxes in *H. pylori oriC*. After incubation with the indicated amounts of DnaA and ATP or ADP, the poriWT plasmid was either treated with P1 nuclease, BglII digested, and resolved by agarose gel electrophoresis (**a**) or modified with DMS and used as a template in PE reactions containing ^32^P-labeled primers E1 (**b**) and E2 (**c**). The protected guanine residues (G) are to the right of the gels. Except for the newly identified box c-ATP DnaA, for which the DNA sequence is presented, the DnaA boxes are marked by continuous lines.

The P1 nuclease assay using poriWT was conducted in the presence of ATP–DnaA and ADP–DnaA (Materials and Methods). The linearized plasmids were subsequently digested with BglII and resolved on agarose gels (Figure 6A).

The results indicate that ATP–DnaA is more efficient at *H. pylori* DUE opening than ADP–DnaA. For DMS footprinting, ATP–DnaA and ADP–DnaA were incubated with the poriWT plasmid. Protection of the DnaA boxes was monitored by PE using primers E1 and E2 (Table S2), which are complementary to the upstream regions of the ts boxes in *oriC2* and the boxes in *oriC1*, respectively. In the presence of ATP, we detected DnaA binding to both ts boxes and observed the DMS hypersensitive region (Figure 6B and Figure S5). Interestingly, ADP–DnaA exhibited decreased affinity for DnaA box ts1 in comparison to ATP–DnaA, whereas the binding of ADP–DnaA to DnaA box ts2 remained unchanged; please note that we could only estimate protection of G2 and G4 of the DnaA ts2 box, but there was no protection of G9 and G8 of the box. Nevertheless, we conclude that DnaA box ts1 (5′-TCATTCCAT-3′) is an ATP–DnaA box. Moreover, we could not detect the DMS hypersensitive region when the poriWT plasmid was incubated with ADP–DnaA (Figure 6B and Figure S5). Lack of DMS hypersensitivity indicates that ADP–DnaA, in the concentration range used, cannot unwind or stabilize ssDNA. This finding corroborates the results of the P1/BglII assay (Figure 6A), which showed that a higher DnaA-ADP concentration than ATP–DnaA was required to unwind *oriC*. The analysis of *H. pylori oriC* allowed us to identify a similar sequence (5′-TTATTCCTT-3′) within the *oriC1* subregion located between DnaA boxes c3 and c4 (Figure 6). The residues between boxes c3 and c4 have been previously shown to be bound by DnaA; however, the detected sequence was not considered a DnaA box [40]. Using DMS footprinting, we confirmed that DnaA binds to this DnaA box but that it does so only as ATP–DnaA, whereas DnaA boxes c2, c3, c4, and c5 are bound irrespective of the nucleotide status of DnaA (Figure 6C and Figure S5). The newly identified box was designated c-ATP DnaA box. The ATP-dependent DnaA boxes’ sequences were assembled to generate a consensus ATP-sensitive binding site (5′-TYATTCCWT-3′). It is noteworthy that the *H. pylori* genome contains 124 in silico identified ATP-dependent DnaA boxes (see Materials and Methods), of which only the two described above are located near high-affinity DnaA boxes.

### 2.4. Mutation of DnaA Box ts1 Is Insufficient for Inhibition of H. pylori OriC Unwinding In Vitro and In Vivo

**Figure 7:**
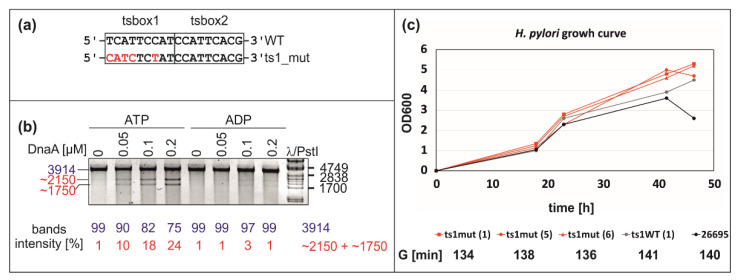
Analysis of the influence of the ts1 box mutation on the functionality of the *oriC* region. (**a**) The sequence of the wild-type and mutated ts1 box is shown. The mutated nucleotide residues are marked in red. (**b**) P1 nuclease analysis of the pori_ts1mut plasmid unwinding by ATP–DnaA and ADP–DnaA. After incubation with the indicated amounts of DnaA and ATP or ADP, the pori_ts1mut plasmid was treated with P1 nuclease, BglII digested, and resolved on 1% agarose gels. The sizes of the λ/PstI DNA ladder bands and the expected DNA restriction fragments (in base pairs) are indicated to the left and right of the gel images, respectively. (**c**) Analysis of *H. pylori* growth under microaerobic conditions. *H. pylori* was inoculated in Brucella broth with an OD_600_ = 0.005 and cultured microaerobically until a stationary phase of growth was reached. The growth curves of the wild-type (ts1WT), and three mutant clones (ts1mut) are shown. Generation times (G) were calculated for the *H. pylori* 26695 wild-type and mutant strains grown in liquid cultures for a time of approximately 0–18 h of growth.

Our results indicated that DnaA box ts1 might be important for the stabilization of the open complex. It was previously shown that DnaA box ts2 is important for DNA unwinding and that DnaA box ts1 is only bound when DnaA interacts with DnaA box ts2 [40] (see also Discussion). To analyze the influences of DnaA box ts1 on DnaA filament formation and DUE unwinding, we examined DnaA’s interactions with the pori_ts1mut plasmid and DUE unwinding by DMS footprinting and P1 nuclease assays, respectively. The pori_ts1mut plasmid contained a randomized DnaA box ts1 (5′-TCATTCCAT-3′ mutated to 5′-CATCTCTAT-3′) (Table S1, Figure 7A).

The P1 analyses indicated that sequence randomization of DnaA box ts1 did not significantly reduce DUE unwinding (Figure 7B and Figure S6A). The pori_ts1mut plasmid was still exclusively unwound by ATP–DnaA but not by ADP–DnaA (Figure 7B). Subsequent PE analysis of P1 nuclease-treated pori_ts1mut plasmid (Figure S6A) showed that DnaA unwinds DNA at the same sequence as in the wild-type ts1. DMS footprinting of the mutated plasmids indicated that the mutation of box ts1 probably affected the interaction between DnaA and ts1mut, and to some extent the interaction between DnaA and ts2; the interactions with c6 DnaA box and DNA looping remained intact (Figure 6 and Figure S10). In the next step, we examined whether the mutation of the ts1 box introduced into a chromosome affects *H. pylori* growth. We compared the growth of three independent clones carrying the same mutation as in the pori_ts1mut vector (Figure 7C) with the growth of control strains: (1) 26695 wild-type strain; (2) ts1_WT, in which a kanamycin cassette was introduced in the same locus as in ts1mut strains, but the ts1 DnaA box was as in the wild-type strain (Figure S7). We did not observe any significant differences in the growth of mutant strains in comparison to both control strains at the logarithmic phase of growth (Figure 7C); all cultures reached similar densities at the stationary phase; ts1_mut strains reached higher final OD600 than the WT and ts1_WT strains. Nonetheless, mutant strains did not accomplish an additional round of duplication when compared to the WT or ts1_WT strains.

Due to the minor changes or the lack of observed changes in orisome formation and *H. pylori* growth, the function of DnaA box ts1 remains ambiguous.

## 3. Discussion

We thoroughly characterized the *oriC2* region of *H. pylori*, determining the roles of the motifs involved in DNA unwinding and stabilization, i.e., the GC-rich sequence, the DnaA-trio, and the AT-rich region. We revealed the putative interplay between DnaA binding to ssDNA and its interaction with DnaA box ts1 upon DNA unwinding. Our results also suggest that ATP is involved in the initiation of *H. pylori* chromosome replication.

### 3.1. DNA Modules in the Vicinity of or Within the H. pylori DUE Region

We showed by KMnO_4_ footprinting that *H. pylori oriC* unwinding begins immediately downstream of the GC-rich sequence and proceeds towards the AT-rich region (Figure 1B and Figure S1). Similarly to the unwinding reaction at *B. subtilis oriC* [38], the DnaA-trio submodule is unwound by DnaA in *H. pylori oriC*. Based on the results of P1/PE assays, we estimated that the unwound DNA is bound by DnaA covering approximately 13–22 nt, including five perfect DnaA-trio repeats (consensus sequence 5′-YTR-3′), and probably also an additional 6 nt encompassing two imperfect repeats (5′-ATT-3′, approximately 22 bases in total). Mutations introduced into the DnaA-trio submodule that affect the sequence of the first five repeats of the motif (poriTrio2-4) prevented unwinding (Figure 2, Figure 3, Figure 4 and Figure 5), probably due to a lack of interaction between DnaA and ssDNA [11]. The mutations also reduced DnaA binding to DnaA box ts1 (see below). Thus, our studies confirmed that the DnaA-trio submodule is essential for the initial unwinding and stabilization of the open complex in bacteria that possess this motif.

The in silico comparison of multiple bacterial *oriC*s (Figure S8) indicates that the length of the DnaA-trio submodule is conserved and that it comprises approximately 5 DnaA-trio repeats. We showed that the elongation of the DnaA-trio submodule by 3 repeats increased the length of the ssDNA oligomer in vitro and did not affect its functionality (Figure 2, Figure 3, Figure 4 and Figure 5). It is unknown whether any further elongation of the DnaA-trio would be tolerated in vitro and how such changes might affect DNA unwinding in vivo. The complementary DnaA-trio 5′-YTD-3′ consensus sequence derived from the 10 DnaA-trio repeats of *B. subtilis* and *H. pylori* DUEs is relatively relaxed (see also Figure 4 in [11]) except for the middle T residue; that residue is highly conserved, suggesting that it may play a pivotal role in DnaA–ssDNA interactions. We show that conservation of this residue is not sufficient for the function of the motif (compare poriWT with poriTrio4—Figure 2, Figure 3, Figure 4 and Figure 5); the sequences of *H. pylori* imperfect DnaA-trio (5′-ATT-3′) are probably also bound by DnaA. Thus, further studies are required to reveal the molecular determinants of the DnaA-trio sequence conservation. The DnaA-trio is found in many but not all bacterial *oriC*s. In *E. coli oriC,* only two scattered (perfect) and/or three clustered (single perfect) DnaA-trio repeats are present, and their role in ssDNA unwinding has not been determined [11,16]. *E. coli* DnaA exhibits a preference for ssDNA 6-mer DnaA-ATP boxes [35] and T-rich sequences [14] (see Introduction). These motifs/sequences are also conserved in some other enterobacterial *oriC* [50]. On the other hand, structural studies of *A. aeolicus* DnaA have shown that nucleotide-specific interactions are not crucial for DnaA and ssDNA interactions [15]. The residues in DnaA corresponding to *E. coli* V211 and K243, which are located within the H and B motifs, respectively, and are important for interactions with ssDNA [15,34], are relatively well conserved in *E. coli, B. subtilis, A. aeolicus*, and *H. pylori* DnaA, whereas residues of the B-motif corresponding to *E. coli* R245 and S246 are less conserved (Figure S9). Whether or not these and other differences in the H/B motifs of diverse bacteria affect the specificity of DnaA–ssDNA sequence recognition remains to be elucidated.

The GC-rich region is the least characterized motif of the bacterial *oriC* region. The sequence rich in GC base pairs was recently shown to be important for the helical instability of DNA because its increased GC content enhances supercoiling-induced DNA melting at the adjacent AT-rich DNA regions [51]. We revealed that the GC-rich sequence is not crucial for DNA unwinding per se, but it is possibly important for maintaining the optimal distance for the interactions between DnaA–dsDNA and ssDNA (see also below). We showed that decreased GC-content (poriG2) or increased length of the GC-rich sequence (poriG3), which possibly changes the distance between DnaA–dsDNA and ssDNA, reduces the stability of DnaA–ts1 box interactions, as in the case of mutants lacking the DnaA-trio, with which the DnaA–ssDNA oligomer cannot be formed (see below). In silico analysis of bacterial *oriC*s (Figure S8) and previous studies [11] indicate that the GC-rich sequence usually encompasses 4 or 5 bp and directly follows R1*_E. coli_*-type DnaA box and precedes the DnaA-trio. However, not all bacteria contain a GC-rich region of the classical type, with *E. coli* being the best example. *E. coli* and related enterobacteria may utilize a distinct type of unwinding and ssDNA stabilization mechanism, partially due to IHF-dependent initiation complex formation arising from the greater distances between the R1*_E. coli_*-type DnaA box and other boxes of the cluster, particularly DnaA R5M, which are required for ssDNA binding [2,16]. Nonetheless, even in *E. coli* and possibly also in related enterobacterial species with highly similar DUE regions [50], the distance (in *E. coli* 13 bps) is more important than the sequence between the R1*_E. coli_*-type DnaA box and the DUE, particularly the R 13-mer, for DnaA’s ability to unwind DNA at the DUE [16,52]. It was shown earlier that even short (1 or 2 bp) deletions or insertions led to drastic reductions of DUE unwinding, whereas sequence changes had only marginal effects [52]. Our observations suggest that the GC-rich sequence ensures correct spacing between the R1*_E. coli_*-type DnaA box and the first DnaA-trio repeat; this spacing might be crucial for the recruitment of DnaA to ssDNA by preventing steric hindrance in a DnaA continuous filamentation model or by ensuring elasticity sufficient to position DnaA-trio in the vicinity of box ts2 in a model of ssDNA recruitment to the dsDNA–DnaA complex.

### 3.2. ATP–DnaA Boxes at H. pylori OriC

We previously noted that DnaA box ts1 (5′-TCATTCCAT-3′) was bound by DnaA only when DnaA interacted with DnaA box ts2 [40]. Thus, ts1 is a relatively weak DnaA box and its binding requires cooperativity with DnaA box ts2. The present results showed that DnaA boxes ts1 and c-ATP (5′-TTATTCCTT-3′) were bound exclusively by ATP–DnaA (Figure 6 and Figure S5). The ATP–DnaA boxes ts1 and c-ATP (consensus sequence 5′-TYATTCCWT-3′) share a highly characteristic feature with weak ATP–DnaA boxes of *E. coli* (5′-TGATCC-3′, 2-7 nt of the 9-mer DnaA box) and *C. crescentus* (5′-KMRTCCCSM-3′) [13,41,53], namely, the presence of a cytosine residue at position 7. In strong DnaA boxes bound by DnaA regardless of the nucleotide form, this position is usually occupied by the adenine residue (e.g., *E. coli* R1/R4 5′-TTATCCACA-3′ and *H. pylori* c2/c3 5′-TCATTCACA-3′). The residue in the 7^th^ position has been shown to be responsible for the ATP–DnaA box phenotype in *E. coli* and *C. crescentus* [41,54]. On the other hand, two amino acid residues, D433 and L438, are important for interaction with the nucleobases at the 7^th^ position of R-type DnaA box in both DNA strands [55,56]. The amino acid residue corresponding to D433 is highly conserved among all classes of bacteria, whereas the residue corresponding to L438 is not [42]. Thus, taking into consideration the nearly universal sequence of the identified ATP–DnaA boxes in different species and the conservation of specific amino acid residues in DnaA, it can be assumed that the observed difference in affinity between ATP–DnaA and ADP–DnaA boxes is caused by interference with hydrogen bond formation between the nucleobases at the 7^th^ position of the DnaA box and the D433 residue in *E. coli* or the corresponding residues in the DnaAs of other species.

Interestingly, both ts1 and c-ATP ATP-boxes are located in putatively important positions at *oriC—*one ATP–DnaA box per each subregion. Each of the subregions is essential for *oriC* function in *H. pylori* [37]. DnaA box c-ATP fills in the c2–c3 and c4–c5 DnaA box arrays at *oriC1*, which then contain 5 DnaA boxes separated by 2 bp, the optimal distance for the filament [19]. ts1 corresponds to DnaA box 6 in *B. subtilis incC* region, and this box was shown to be indispensable for *B. subtilis oriC* activity [11,39]. In vitro *H. pylori oriC* unwinding depends on ATP (Figure 6A). However, whether or not ATP-ts DnaA boxes are involved in the regulation of DnaA assembly in vivo remains to be elucidated (see also below). It is noteworthy that *H. pylori* lacks Hda, which in Gammaproteobacteria (e.g., *E. coli* and *C. crescentus*) controls the ATP–DnaA level during the cell cycle [57,58]. Nevertheless, the possible regulation of DnaA activity by ATP or ADP in vivo is an open question. Since ts1 is also topology-sensitive, its binding is presumably also regulated by topology in vivo. It was recently shown that topology-sensitive binding of DnaA to *oriC* regulates chromosome replication in *B. subtilis,* suggesting that this mechanism of initiation regulation might be more common in bacteria than has been considered thus far [59].

### 3.3. Intriguing Role of the ts1 DnaA Box

ts1 corresponds to *B. subtilis* box 6 or *E. coli* R5M box, both of which are crucial for origin unwinding. Moreover, the ts1–ts2 DnaA box arrangement resembles *B. subtilis* 6 and 7 boxes; i.e., there is no space between boxes ts1 and ts2, and boxes 6 and 7 overlap. However, the ts1 mutation did not affect the efficiency of DNA unwinding in vitro or the initial unwinding site (Figure 7). A similar situation was observed in *B. subtilis*, in which the DnaA box 6 was not essential for DnaA filamentation on ssDnaA-trio as long as DnaA box 7 was present [11]. However, in contrast to the in vitro results, only DnaA box 6 was essential for *B. subtilis* viability. We can also see some correlation if we compare the *H. pylori* ts DnaA boxes with *E. coli* boxes R1 and R5M. These two boxes were recently shown to be crucial for ssDNA recruitment to the DnaA–dsDNA complex [34]. ts2 corresponds to the strong, ATP-independent *E. coli* DnaA box R1, and ts1 corresponds to DnaA box R5M. Although R5M is a weak ATP–DnaA box, it seems to be more important for ssDNA binding than DnaA box R1 [34]. Moreover, it also seems to be more important in vivo than DnaA box R1, probably due to its important role in open complex stabilization. Both DnaA boxes R1 and R5M are crucial for the in vivo functionality of *E. coli oriC* [60].

Our results, however, indicate that mutation of DnaA ts1 box, which changed not only its ATP-relevant sequence (CC→CT at position 5–6 of the 9-mer sequence; see below) but also the critical nucleotides of the consensus *H. pylori* DnaA box (4th and 6–8th positions of the 9-mer: TTCAC→CTCTA, [42]), did not disturb the origin activity in vitro; in vivo, the mutation of ts1 box, unlike the mutation of *B. subtilis* DnaA box 6, was not lethal, nor did it cause any major growth defects (Figure 7 and Figure S6). However, we cannot unequivocally say that DnaA did not bind the ts1mut DnaA box because DMS footprinting detects only limited sites at dsDNA (i.e., guanine and adenine residues; it does not detect interactions with a phosphate backbone [48]). Thus, DnaA may still bind the ts1_mut DnaA box, and hence the unwinding of pori_ts1mut plasmid and the growth of *H. pylori* ts1mut strain were not disturbed. No other footprinting techniques can be used to verify DnaA–ts1 interactions because they require the usage of modifying agents that destroy DNA superhelical DNA topology (DNaseI, copper-phenanthroline). It should also be noted that by mutating ts1, we also disturbed the HP1021 box, which overlaps with the ts1 DnaA box; HP1021 competes with DnaA to bind to DNA [49]. Thus, it is possible that by mutating the ts1 DnaA box, we also affected another level of *H. pylori* chromosome replication regulation.

It is worth noting that origins, especially DUE regions, are highly similar in *H. pylori* and *B. subtilis*. DUE-proximal *H. pylori* ts1 and ts2 DnaA boxes and *B. subtilis* DnaA boxes 6 and 7 are fused or overlap, which is not typical for DnaA box scaffolds (please compare right and left *E. coli* DnaA pentamers; see Introduction and [33]). Both origins contain an array of DnaA-trio motifs downstream of DUE-proximal DnaA boxes (*H. pylori* ts1 and ts2; *B. subtilis* 6 and 7), separated by an GC-rich region. However, *B. subtilis* DnaA box 6, corresponding to *H. pylori* ts1, is essential for *B. subtilis* viability; mutation of ts1 did not preclude *H. pylori* chromosome replication. This suggests that either the mutagenesis was inefficient and DnaA could still bind the ts1mut DnaA box or that the mode of DnaA oligomer assembly is different in both species despite similar structural elements of the DUE region. Further studies, especially more comprehensive ts1/ts2 box mutagenesis, are required to reveal the exact role of ts1 and ts2 in *H. pylori* orisome assembly and DNA unwinding; however, the limited number of available techniques for studying DnaA interactions with supercoiled DNA makes these studies challenging.

### 3.4. Putative Interdependent Interaction between ts1 and ssDNA upon DNA Unwinding

**Figure 8:**
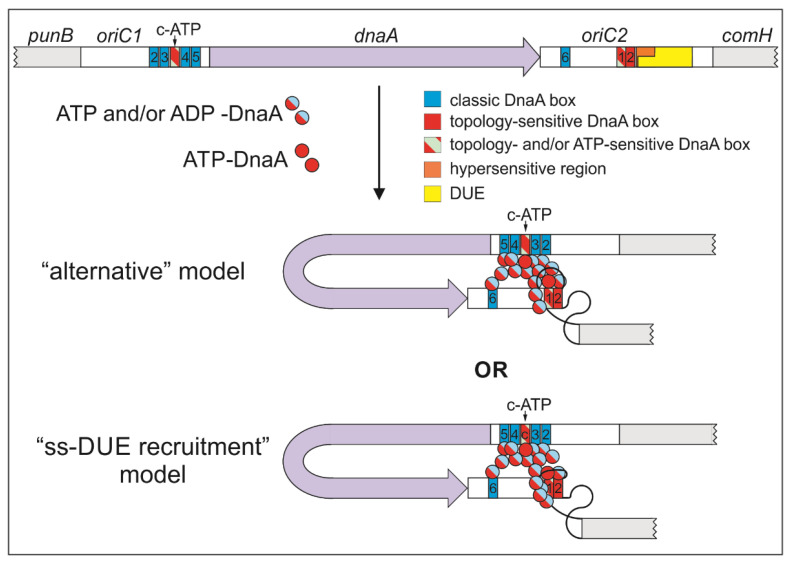
Schematic representation of possible *H. pylori* orisome structures on bipartite *oriC*. Boxes c-ATP and ts1 are bound exclusively by ATP–DnaA, whereas both DnaA forms, ADP-bound and ATP-bound, interact with classic DnaA boxes. The nucleotide status of the DnaA (ATP or ADP) forming the complete oligomer is unknown. The binding of DnaA to dsDNA leads to DNA unwinding. DnaA bound to ssDNA stabilizes the interaction of the protein with DnaA box ts1 either by recruitment of additional DnaA molecules (“alternative” model) or by simultaneous ssDNA and dsDNA binding (“ss-DUE recruitment” model). Whether or not there is additional stabilization/orientation of ssDNA–DnaA oligomer by DnaA bound to *oriC1* of *H. pylori* is also an open question.

The inability of DnaA to unwind DNA after deletion of the DnaA-trios (poriTrio2–4) precluded DnaA’s interaction with DnaA box ts1, suggesting an interdependency of the interactions of DnaA with dsDNA and ssDNA (Figure 2, Figure 3, Figure 4 and Figure 5 and Figure S4). The distance between DnaA bound to the ts boxes and the opened ssDNA changed in poriG2 and poriG3, and this reduced the stability of DnaA-DnaA box ts1 interactions (Figure 3, Figure 4 and Figure 5 and Figure S4), corroborating our results that ssDNA interacts with DnaA–dsDNA. Our results contribute to the discussion of DnaA assembly models for DNA melting. However, we cannot unequivocally determine whether the DnaA–ts1 complex is stabilized by the interaction between two different DnaA molecules individually bound to DnaA box ts1 and ssDNA, as suggested by the “alternative” model of DnaA filamentation (Figure 8, [34]; see also Introduction), or by simultaneous binding of the same DnaA molecule to box ts1 and ssDNA, as suggested by the “ssDUE-recruitment” model [33,34].

The continuous filamentation model was supported by studies on *B. subtilis* orisome assembly [11,39]. Based on crosslinking analysis, it was proposed that the DnaA filament is formed on dsDNA DnaA boxes 6 and 7 and that the DnaA bound to DnaA box 7 then invades the dsDnaA-trio, unwinds the DNA, and allows DnaA filament to develop on the ssDNA-trios. It should be noted that these studies were conducted using short, linear DNA fragments; thus, the results might not reflect interactions with full-length supercoiled *oriC* or the interactions that occur during in vivo orisome formation. The structures of *oriC* regions and orisomes differ among bacterial species [61], especially when *H. pylori/B. subtilis* bipartite-type origins are compared to *E. coli* monopartite *oriC,* or when IHF-dependent orisomes are compared to IHF-independent ones [34]. Thus, the mechanism of ssDNA binding by DnaA with regard to the dsDNA–DnaA filament might vary for different types of origins. Further studies are required to characterize better the structure of the initiation complex upon DNA unwinding.

## 4. Materials and Methods

### 4.1. In Silico Analysis

The SIDD prediction of DNA stability was calculated as described previously [42] using the SIST software package [62]. Identification of ATP-dependent DnaA boxes in *H. pylori* was conducted using Pattern locator [63] and a consensus sequence of the ATP-sensitive binding site (5′-TYATTCCWT-3′).

### 4.2. Materials and Culture Conditions

The strains, plasmids, and proteins used in this work are listed in Table S1. The primers used in this study are listed in Table S2. *E. coli* DH5α and MC1061 [64,65] were grown at 37 °C on solid or in liquid lysogeny broth (LB), supplemented with 100 µg/mL ampicillin (Carl Roth GmbH, Karlsruhe, Germany) when necessary. *H. pylori* 26695 [66] or derivative mutant strains were cultivated at 37 °C under microaerobic conditions (5% O_2_, 10% CO_2_, and 85% N_2_) generated by the jar evacuation-replacement method. *H. pylori* plate cultures were grown on Columbia blood agar base medium (Oxoid, Thermo Fisher Scientific, Waltham, MA, USA) supplemented with 8% defibrinated horse blood (ProAnimali, Wrocław, Poland) (CBA-B). The liquid cultures were prepared in Brucella broth (Becton Dickinson, NJ, USA) containing 10% fetal calf serum (Biowest, Nuaillé, France). All *H. pylori* cultures were supplemented with an antibiotic mix [67]; when necessary, kanamycin (Carl Roth GmbH, Karlsruhe, Germany) was added to 15 μg/mL. The growth of the liquid cultures was monitored by measuring the optical density at 600 nm (OD_600_). *E. coli* DH5α and BL21 were used for cloning and recombinant protein synthesis, respectively, while *E. coli* MC1061 was used to propagate plasmids used to transform *H. pylori*. Plasmids and DNA fragments were purified using a GeneJET Gel Extraction Kit, GeneJET Plasmid Miniprep Kit, GeneJET Plasmid Midiprep Kit (Thermo Scientific, Waltham, MA, USA) or Plasmid Midi AX (A&A Biotechnology, Gdańsk, Poland).

### 4.3. Protein Expression and Purification

The purification of untagged or strep-tagged *H. pylori* DnaA was described previously [68,69,70]. The use of a specific form of protein (untagged DnaA or Strep-tagged DnaAStrep) is marked in each figure. To investigate ATP/ADP influence on DNA binding, the DnaA protein was pre-incubated at 37 °C for 10 min to ensure hydrolysis of residual ATP and reaction mixtures supplemented with ATP or ADP (Carl Roth GmbH, Karlsruhe, Germany) were subsequently prepared. 6HisHU, cloned into a pET151/D-TOPO derivative ([71] and Supplementary Methods), was isolated as described in Supplementary Methods.

### 4.4. DMS Footprinting

In vitro DNA modification was performed as described previously [40,44,72] using DnaA at a concentration range of 0.4–1.6 µM. DMS-methylated poriWT plasmid and mutated derivatives were used as templates in primer extension (PE) reactions containing the appropriate primers (see Table S2). The gels were scanned using a Typhoon FLA 9500 (GE Healthcare, Chicago, IL, USA) and analyzed using ImageQuant (GE Healthcare, Chicago, IL, USA) and ImageJ (https://imagej.nih.gov/ij/ accessed on 13 June 2021).

### 4.5. P1 Nuclease Assay

The P1 nuclease assay was conducted as described [37]. Reaction mixtures contained 300 ng of plasmid DNA (approximately 10 nM) and DnaA (up to 0.5 µM) in a total volume of 15 µL; 6HisHU was added when indicated. P1 activity was analyzed by restriction enzyme digestion (P1/BglII) and 1% agarose gel separation or PE analysis (P1/PE). The gels were scanned using a GelDoc Xr+ Imaging System (Bio-Rad, Hercules, CA, USA). When indicated, the gels were analyzed densitometrically using ImageLab (BioRad, Hercules, CA, USA). DNA bands were quantified and presented as a percentage of the intensity of total plasmid resolved in each lane.

### 4.6. Primer Extension (PE) Reactions

The DNA modifications introduced by DMS, KMnO_4_, or P1 nuclease (Sigma, Merck KGaA, Darmstadt, Germany) were monitored by PE analysis. The reaction conditions, mixture separation, and product visualization were described previously [42]. The gels were scanned using a Typhoon FLA 9500 (GE Healthcare, Chicago, IL, USA) and analyzed using ImageQuant (GE Healthcare, Chicago, IL, USA) and ImageJ (https://imagej.nih.gov/ij/ accessed on 13 June 2021).

## Data Availability

The data that support the findings of this study are available from the corresponding author, (A.Z.-P.), upon reasonable request.

## Supplementary Materials

The following are available online at https://www.mdpi.com/article/10.3390/ijms22126643/s1. Supplementary Materials and Methods. Figure S1: Precise mapping of the *H. pylori* 5′ DUE border. Figure S2: In silico analysis of stress-induced duplex destabilization (SIDD) in plasmids with introduced mutations. Figure S3: HU influence on DnaA-dependent *oriC* unwinding. Figure S4: Densitometric analysis supplementing DMS analysis of DUE submodules mutations (Figure 3). Figure S5: Densitometric analysis supplementing the identification of the ATP-dependent DnaA boxes (Figure 6). Figure S6: Analysis of the influence of the ts1 box mutation on the functionality of the *oriC* region. Figure S7: The mutagenesis strategy used to mutate the ts1 DnaA box on the *H. pylori* chromosome. Figure S8: DnaA-trio motifs in the *oriC* replication origins of Epsilonproteobacteria (a) and bacteria from more than 20 different phyla (b). Figure S9: Alignment of the amino acid sequences of DnaA of *H. pylori* (HP), *A. aeolicus* (AA), *E. coli* (EC), and *B. subtilis* (BS). Figure S10: Representative electron micrographs of selected mutated plasmids presenting structures formed by DnaA interactions with suborigins. Table S1: Strains, plasmids, and proteins used in this work. Table S2. Primers used in this work.

## References

[1] Katayama T., Ozaki S., Keyamura K., Fujimitsu K. (2010). Regulation of the replication cycle: Conserved and diverse regulatory systems for DnaA and *oriC*. Nat. Rev. Microbiol..

[2] Leonard A.C., Grimwade J.E. (2015). The orisome: Structure and function. Front. Microbiol..

[3] Zawilak-Pawlik A., Nowaczyk M., Zakrzewska-Czerwińska J. (2017). The Role of the N-Terminal Domains of Bacterial Initiator DnaA in the Assembly and Regulation of the Bacterial Replication Initiation Complex. Genes.

[4] Leonard A.C., Méchali M. (2013). DNA replication origins. Cold Spring Harb. Perspect. Biol..

[5] Duderstadt K.E., Berger J.M. (2013). A structural framework for replication origin opening by AAA+ initiation factors. Curr. Opin. Struct. Biol..

[6] Erzberger J.P., Mott M.L., Berger J.M. (2006). Structural basis for ATP-dependent DnaA assembly and replication-origin remodeling. Nat. Struct. Mol. Biol..

[7] Kowalski D., Eddy M.J. (1989). The DNA unwinding element: A novel, cis-acting component that facilitates opening of the *Escherichia coli* replication origin. EMBO J..

[8] Bell S.P., Kaguni J.M. (2013). Helicase loading at chromosomal origins of replication. Cold Spring Harb. Perspect. Biol..

[9] Kaguni J.M. (2011). Replication initiation at the *Escherichia coli* chromosomal origin. Curr. Opin. Chem. Biol..

[10] Ozaki S., Katayama T. (2012). Highly organized DnaA-*oriC* complexes recruit the single-stranded DNA for replication initiation. Nucleic Acids Res..

[11] Richardson T.T., Harran O., Murray H. (2016). The bacterial DnaA-trio replication origin element specifies single-stranded DNA initiator binding. Nature.

[12] Ozaki S., Katayama T. (2009). DnaA structure, function, and dynamics in the initiation at the chromosomal origin. Plasmid.

[13] Kaguni J.M. (2006). DnaA: Controlling the initiation of bacterial DNA replication and more. Annu. Rev. Microbiol..

[14] Ozaki S., Kawakami H., Nakamura K., Fujikawa N., Kagawa W., Park S.-Y., Yokoyama S., Kurumizaka H., Katayama T. (2008). A common mechanism for the ATP-DnaA-dependent formation of open complexes at the replication origin. J. Biol. Chem..

[15] Duderstadt K.E., Chuang K., Berger J.M. (2011). DNA stretching by bacterial initiators promotes replication origin opening. Nature.

[16] Katayama T., Kasho K., Kawakami H. (2017). The DnaA Cycle in *Escherichia coli*: Activation, Function and Inactivation of the Initiator Protein. Front. Microbiol..

[17] Bramhill D., Kornberg A. (1988). Duplex opening by dnaA protein at novel sequences in initiation of replication at the origin of the *E. coli* chromosome. Cell.

[18] Skarstad K., Katayama T. (2013). Regulating DNA replication in bacteria. Cold Spring Harb. Perspect. Biol..

[19] Rozgaja T.A., Grimwade J.E., Iqbal M., Czerwonka C., Vora M., Leonard A.C. (2011). Two oppositely oriented arrays of low-affinity recognition sites in *oriC* guide progressive binding of DnaA during *Escherichia coli* pre-RC assembly. Mol. Microbiol..

[20] Speck C., Weigel C., Messer W. (1999). ATP- and ADP-DnaA protein, a molecular switch in gene regulation. EMBO J..

[21] Miller D.T., Grimwade J.E., Betteridge T., Rozgaja T., Torgue J.J.-C., Leonard A.C. (2009). Bacterial origin recognition complexes direct assembly of higher-order DnaA oligomeric structures. Proc. Natl. Acad. Sci. USA.

[22] McGarry K.C., Ryan V.T., Grimwade J.E., Leonard A.C. (2004). Two discriminatory binding sites in the *Escherichia coli* replication origin are required for DNA strand opening by initiator DnaA-ATP. Proc. Natl. Acad. Sci. USA.

[23] Grimwade J.E., Rozgaja T.A., Gupta R., Dyson K., Rao P., Leonard A.C. (2018). Origin recognition is the predominant role for DnaA-ATP in initiation of chromosome replication. Nucleic Acids Res..

[24] Leonard A.C., Rao P., Kadam R.P., Grimwade J.E. (2019). Changing perspectives on the role of DnaA-ATP in orisome function and timing regulation. Front. Microbiol..

[25] Kawakami H., Keyamura K., Katayama T. (2005). Formation of an ATP-DnaA-specific initiation complex requires DnaA Arginine 285, a conserved motif in the AAA+ protein family. J. Biol. Chem..

[26] Kubota T., Katayama T., Ito Y., Mizushima T., Sekimizu K. (1997). Conformational transition of DnaA protein by ATP: Structural analysis of DnaA protein, the initiator of *Escherichia coli* chromosome replication. Biochem. Biophys. Res. Commun..

[27] Sekimizu K., Bramhill D., Kornberg A. (1987). ATP activates dnaA protein in initiating replication of plasmids bearing the origin of the *E. coli* chromosome. Cell.

[28] Jameson K.H., Wilkinson A.J. (2017). Control of Initiation of DNA Replication in *Bacillus subtilis* and *Escherichia coli*. Genes.

[29] Merrikh H., Grossman A.D. (2011). Control of the replication initiator DnaA by an anti-cooperativity factor. Mol. Microbiol..

[30] Patel M.J., Bhatia L., Yilmaz G., Biswas-Fiss E.E., Biswas S.B. (2017). Multiple conformational states of DnaA protein regulate its interaction with DnaA boxes in the initiation of DNA replication. Biochim. Biophys. Acta Gen. Subj..

[31] Duderstadt K.E., Mott M.L., Crisona N.J., Chuang K., Yang H., Berger J.M. (2010). Origin remodeling and opening in bacteria rely on distinct assembly states of the DnaA initiator. J. Biol. Chem..

[32] Noguchi Y., Sakiyama Y., Kawakami H., Katayama T. (2015). The Arg Fingers of Key DnaA Protomers Are Oriented Inward within the Replication Origin *oriC* and Stimulate DnaA Subcomplexes in the Initiation Complex. J. Biol. Chem..

[33] Shimizu M., Noguchi Y., Sakiyama Y., Kawakami H., Katayama T., Takada S. (2016). Near-atomic structural model for bacterial DNA replication initiation complex and its functional insights. Proc. Natl. Acad. Sci. USA.

[34] Sakiyama Y., Kasho K., Noguchi Y., Kawakami H., Katayama T. (2017). Regulatory dynamics in the ternary DnaA complex for initiation of chromosomal replication in *Escherichia coli*. Nucleic Acids Res..

[35] Speck C., Messer W. (2001). Mechanism of origin unwinding: Sequential binding of DnaA to double- and single-stranded DNA. EMBO J..

[36] Kato J.I., Katayama T. (2001). Hda, a novel DnaA-related protein, regulates the replication cycle in *Escherichia coli*. EMBO J..

[37] Donczew R., Weigel C., Lurz R., Zakrzewska-Czerwińska J., Zawilak-Pawlik A. (2012). *Helicobacter pylori oriC*—the first bipartite origin of chromosome replication in Gram-negative bacteria. Nucleic Acids Res..

[38] Krause M., Rückert B., Lurz R., Messer W. (1997). Complexes at the replication origin of *Bacillus subtilis* with homologous and heterologous DnaA protein. J. Mol. Biol..

[39] Richardson T.T., Stevens D., Pelliciari S., Harran O., Sperlea T., Murray H. (2019). Identification of a basal system for unwinding a bacterial chromosome origin. EMBO J..

[40] Donczew R., Mielke T., Jaworski P., Zakrzewska-Czerwińska J., Zawilak-Pawlik A. (2014). Assembly of *Helicobacter pylori* initiation complex is determined by sequence-specific and topology-sensitive DnaA-*oriC* interactions. J. Mol. Biol..

[41] Taylor J.A., Ouimet M.C., Wargachuk R., Marczynski G.T. (2011). The *Caulobacter crescentus* chromosome replication origin evolved two classes of weak DnaA binding sites. Mol. Microbiol..

[42] Jaworski P., Donczew R., Mielke T., Thiel M., Oldziej S., Weigel C., Pawlik A.M. (2016). Unique and universal features of Epsilonproteobacterial origins of chromosome replication and DnaA-DnaA box interactions. Evol. Genomic Microbiol..

[43] Spicuglia S., Kumar S., Chasson L., Payet-Bornet D., Ferrier P. (2004). Potassium permanganate as a probe to map DNA-protein interactions in vivo. J. Biochem. Biophys. Methods.

[44] Sasse-Dwight S., Gralla J.D. (1991). Footprinting protein-DNA complexes in vivo. Methods Enzymol..

[45] Bui C.T., Rees K., Cotton R.G.H. (2003). Permanganate oxidation reactions of DNA: Perspective in biological studies. Nucleosides. Nucleotides Nucleic Acids.

[46] Wells S.E., Hughes J.M., Igel A.H., Ares M. (2000). Use of dimethyl sulfate to probe RNA structure in vivo. Methods Enzymol..

[47] Hwang D.S., Kornberg A. (1992). Opening of the replication origin of *Escherichia coli* by DnaA protein with protein HU or IHF. J. Biol. Chem..

[48] Lawley P.D., Brookes P. (1963). Further studies on the alkylation of nucleic acids and their constituent nucleotides. Biochem. J..

[49] Donczew R., Makowski Ł., Jaworski P., Bezulska M., Nowaczyk M., Zakrzewska-Czerwińska J., Zawilak-Pawlik A. (2015). The atypical response regulator HP1021 controls formation of the *Helicobacter pylori* replication initiation complex. Mol. Microbiol..

[50] Zyskind J.W., Cleary J.M., Brusilow W.S., Harding N.E., Smith D.W. (1983). Chromosomal replication origin from the marine bacterium Vibrio harveyi functions in *Escherichia coli*: *oriC* consensus sequence. Proc. Natl. Acad. Sci. USA.

[51] Vlijm R.V.D., Torre J., Dekker C. (2015). Counterintuitive DNA Sequence Dependence in Supercoiling-Induced DNA Melting. PLoS ONE.

[52] Hsu J., Bramhill D., Thompson C.M. (1994). Open complex formation by DnaA initiation protein at the *Escherichia coli* chromosomal origin requires the 13-mers precisely spaced relative to the 9-mers. Mol. Microbiol..

[53] Leonard A.C., Grimwade J.E. (2011). Regulation of DnaA assembly and activity: Taking directions from the genome. Annu. Rev. Microbiol..

[54] Grimwade J.E., Torgue J.J.-C., McGarry K.C., Rozgaja T., Enloe S.T., Leonard A.C. (2007). Mutational analysis reveals *Escherichia coli oriC* interacts with both DnaA-ATP and DnaA-ADP during pre-RC assembly. Mol. Microbiol..

[55] Fujikawa N., Kurumizaka H., Nureki O., Terada T., Shirouzu M., Katayama T., Yokoyama S. (2003). Structural basis of replication origin recognition by the DnaA protein. Nucleic Acids Res..

[56] Tsodikov O.V., Biswas T. (2011). Structural and thermodynamic signatures of DNA recognition by *Mycobacterium tuberculosis* DnaA. J. Mol. Biol..

[57] Katayama T., Kubota T., Kurokawa K., Crooke E., Sekimizu K. (1998). The initiator function of DnaA protein is negatively regulated by the sliding clamp of the *E. coli* Chromosomal replicase. Cell.

[58] Collier J., Shapiro L. (2009). Feedback control of DnaA-mediated replication initiation by replisome-associated HdaA protein in *Caulobacter*. J. Bacteriol..

[59] Samadpour A.N., Merrikh H. (2018). DNA gyrase activity regulates DnaA-dependent replication initiation in *Bacillus subtilis*. Mol. Microbiol..

[60] Langer U., Richter S., Roth A., Weigel C., Messer W. (1996). A comprehensive set of DnaA-box mutations in the replication origin, *oriC*, of *Escherichia coli*. Mol. Microbiol..

[61] Wolański M., Donczew R., Zawilak-Pawlik A., Zakrzewska-Czerwińska J. (2014). *oriC*-encoded instructions for the initiation of bacterial chromosome replication. Front. Microbiol..

[62] Zhabinskaya D., Madden S., Benham C.J. (2015). SIST: Stress-induced structural transitions in superhelical DNA. Bioinformatics.

[63] Mrázek J., Xie S. (2006). Pattern locator: A new tool for finding local sequence patterns in genomic DNA sequences. Bioinformatics.

[64] Hanahan D. (1983). Studies on transformation of *Escherichia coli* with plasmids. J. Mol. Biol..

[65] Casadaban M.J., Cohen S.N. (1980). Analysis of gene control signals by DNA fusion and cloning in *Escherichia coli*. J. Mol. Biol..

[66] Tomb J.F., White O., Kerlavage A.R., Clayton R.A., Sutton G.G., Fleischmann R.D., Ketchum K.A., Klenk H.P., Gill S., Dougherty B.A. (1997). The complete genome sequence of the gastric pathogen *Helicobacter pylori*. Nature.

[67] Contreras M., Thiberge J.-M., Mandrand-Berthelot M.-A., Labigne A. (2003). Characterization of the roles of NikR, a nickel-responsive pleiotropic autoregulator of *Helicobacter pylori*. Mol. Microbiol..

[68] Zawilak A., Durrant M.C., Jakimowicz P., Backert S., Zakrzewska-Czerwińska J. (2003). DNA binding specificity of the replication initiator protein, DnaA from *Helicobacter pylori*. J. Mol. Biol..

[69] Zawilak-Pawlik A., Donczew R., Szafrański S., Mackiewicz P., Terradot L., Zakrzewska-Czerwińska J. (2011). DiaA/HobA and DnaA: A Pair of Proteins Co-evolved to Cooperate During Bacterial Orisome Assembly. J. Mol. Biol..

[70] Nowaczyk-Cieszewska M., Zyla-Uklejewicz D., Noszka M., Jaworski P., Mielke T., Zawilak-Pawlik A.M. (2020). The role of *Helicobacter pylori* DnaA domain I in orisome assembly on a bipartite origin of chromosome replication. Mol. Microbiol..

[71] Natrajan G., Hall D.R., Thompson A.C., Gutsche I., Terradot L. (2007). Structural similarity between the DnaA-binding proteins HobA (HP1230) from *Helicobacter pylori* and DiaA from *Escherichia coli*. Mol. Microbiol..

[72] Cassler M.R., Grimwade J.E., Leonard A.C. (1995). Cell cycle-specific changes in nucleoprotein complexes at a chromosomal replication origin. EMBO J..

