# Supplementary material for: Putative Cooperative ATP–DnaA Binding to Double-Stranded DnaA Box and Single-Stranded DnaA-Trio Motif upon Helicobacter pylori Replication Initiation Complex Assembly
Source: Int J Mol Sci. 2021 Jun 21;22(12):6643. doi: 10.3390/ijms22126643 (PMC8235120; doi:10.3390/ijms22126643)
Supplement: Supplementary file 1 [file ijms-22-06643-s001.zip › ijms-1255930-supplementary.pdf]

SUPPLEMENTARY MATERIALS

Putative cooperative ATP-DnaA binding to double-stranded DnaA box and single-stranded DnaA-trio motif upon *Helicobacter pylori* replication-initiation-complex assembly

SUPPLEMENTARY TABLES

Table S1. Strains, plasmids, and proteins used in this work.

**Strain/plasmid/ protein**	**Genotype/feature**	**Reference/ source**
**Strains**		
E. coli		
DH5α	*supE44, hsdR17, recA1, endA1, gyrA1, gyrA96, thi-1, relA1*	[64]
BL21	B F- *dcm ompT hsdS*(rB- mB)*gal*	GE Healthcare
MC1061	F *araD139* (*ara-leu*)*7696 galE15 galK16*, (*lacX74*) *rpsL hsdR2 mcrA mcrB1*	[65]
H. pylori		
26695	Wild-type strain	[66]
26695 26695Δ1021::*aph-3*	*H. pylori* 26695 with HP1021 exchanged to *aph*-3 cassette	[49]
ts1mut	*H. pylori* 26695 with ts1 DnaA box (5′-TCATTCCAT-3′) mutated to 5′-CATCTCTAT-3′ and the *aphA-3* cassette inserted between *ori2* and *comH* (HP1527)	This work
ts1WT	*H. pylori* 26695 with the *aphA-3* cassette inserted between *ori2* and *comH* (HP1527), as in the ts1mut-*aphA-3*	This work
**Plasmids**		
poriWT	A pOC170 derivative lacking *E. coli oriC* and containing a DNA fragment of the *H. pylori* 26695 genome encompassing *oriC1* and *oriC2* subregions and the *dnaA* gene. In the original work, the plasmid was referred to as pori1ori2.	[37]
pET28a(+) ΔHistag*hpdnaA*	A pET28a derivative containing the *H. pylori dnaA* gene; used for expression of untagged DnaA protein	[68]
pET151-Nterm	a vector expressing the N-terminal fragment of DnaA (DnaAI–II, residues1–112) fused to a TEV-cleavable histidine tag	[71]

pET151-HU	a vector expressing the *H. pylori* HU protein fused to a TEV-cleavable histidine tag	This work
pori_ts1mut	poriWT derivative with randomized ts1 DnaA box; constructed using the primer pairs P1/P10 and P2/P9	This work
pori_ts1mut-*aphA3*	pori_ts1mut derivative with *aphA-3* cassette and the 5′ region of *comH* gene introduced downstream of *oriC2*; constructed using the primer pairs P33/P35 and P34/P36 (Figure S6)	This work
pori_ts1WT-*aphA3*	pori_WT derivative with *aphA-3* cassette and the 5′ region of *comH* gene introduced downstream of *oriC2*; constructed using the primer pairs P33/P35 and P34/P36 (Figure S6)	This work
poriG1	poriWT derivative with a mutation between the DnaA box ts2 and DUE; constructed using the primer pairs P1/P12 and P2/P11 (Figure 2)	This work
poriG2	poriWT derivative with a mutation between the DnaA box ts2 and DUE that alters the region's GC content; constructed using the primer pairs P1/P32 and P2/P31 (Figure 2)	This work
poriG3	poriWT derivative with additional cytosines between the DnaA box ts2 and DUE; constructed using the primer pairs P1/P24 and P2/P23 (Figure 2)	This work
poriG4	poriWT derivative with an additional DnaA trio instead of triple cytosines; constructed using the primer pairs P1/P26 and P2/P25 (Figure 2)	This work
poriTrio1	poriWT derivative with three additional DnaA trio repeats; constructed using the primer pairs P1/P28 and P2/P27 (Figure 2)	This work
poriTrio2	poriWT derivative with the modified orientation of the DnaA trio motif; constructed using the primer pairs P1/P14 and P2/P13 (Figure 2)	This work
poriTrio3	poriWT derivative with strand exchange within the DnaA trio motif; constructed using the primer pairs P1/P17, P2/P18, P15/P20 and P16/P19 (Figure 2)	This work
poriTrio4	poriWT derivative with randomization of the DnaA trio motif; constructed using the primer pairs P1/P22 and P2/P21 (Figure 2)	This work
poriAT1	poriWT derivative with deletion of the AT-rich sequence downstream of the DnaA trio; constructed using the primer pairs P1/P30 and P2/P29 (Figure 2)	This work
**Proteins**		

DnaA	Recombinant, untagged *H. pylori* DnaA protein	[37]
DnaAStrep	Recombinant, strep-tagged *H. pylori* DnaA protein	[70]
6HisHU	Recombinant, his-tagged *H. pylori* HU protein	This work

Table S2. Primers used in this work.

Name	5′ – 3′ sequence	Application
**pori plasmids**		
P1	GCGAATTCGAGCTCGGTACCCCGCAA	See Materials and Methods<br>Table S1
P2	GCCTGCAGGGTTTAGTAAAAGTCATAAATA	
P9	AAGCTTTTTATTGTTCATCTCTATCCATTCACGCCCCTA	
P10	TAGGGGCGTGAATGGATAGAGATGAACAATAAAAAGCTT	
P11	TCCATCCATTCACGGGGCTACTACTGTTACT	
P12	AGTAACAGTAGTAGCCCCGTGAATGGATGGA	
P13	ATCCATTCACGCCCCATTGTCATCATCTAATTATTATTAAT	
P14	ATTAATAATAATTAGATGATGACAATGGGGCGTGAATGGAT	
P15	TTTCATTCCATCCATTCACGGGGGATGATGACAATGATTAATAAT	
P16	TGGATTATAGCTTAAAAAAACCAAATCATTTTCAGTATTTATCC	
P17	CGTGAATGGATGGAATGAAACAA	
P18	TTTTTTTAAGCTATAATCCAGGGGA	
P19	TTAATTTCACAATGAATAGATATTGGATAAATACTGAAAATGATTG	
P20	TATCTATTCATTGTGAAATTAATTATTATTAATCATTGTCATCATCCC	
P21	TCCATTCACGCCCCTGATCATACTTCTAATTATTATTAAT	
P22	ATTAATAATAATTAGAAGTATGATCAGGGGCGTGAATGGA	
P23	TGTTTCATTCCATCCATTCACGCCCCCCCTACTACTGTTACTAATTATT	
P24	AATAATTAGTAACAGTAGTAGGGGGGGCGTGAATGGATGGAATGAAACA	
P25	TGTTTCATTCCATCCATTCACGCTACTACTACTGTTACTAATTATT	
P26	AATAATTAGTAACAGTAGTAGTAGCGTGAATGGATGGAATGAAACA	
P27	CATTCCATCCATTCACGCCCCTACTACTACTACTACTGTTACTAATTATTAT	
P28	TTAATAATAATTAGTAACAGTAGTAGTAGTAGTAGGGGCGTGAATGGATG	
P29	CACGCCCCTACTACTGTTACTAACTTATCTATAACCTATTTA	
P30	TAAATAGGTTATAGATAAGTTAGTAACAGTAGTAGGGGCGTG	
P31	TGTTTCATTCCATCCATTCACGAAACTACTACTGTTACTAATTATT	
P32	AATAATTAGTAACAGTAGTAGTTTCGTGAATGGATGGAATGAAACA	
***H. pylori* mutagenesis**		
P33	AACTGCAGTGACTAACTAGGAGGAATAAATG	Figures 7 and S7
P34	GAATTGTTTTAGTACCTGGAGGGAATAATGAAAAAATCCCTTTGTCTGTCT	
P35	AGACAGACAAAGGGATTTTTTCATTATTCCCTCCAGGTACTAAAACAATTC	
P36	CGGTGCGGGTGCGACTCTTTCAATTC	
P37	GTATCTTTTACGCAGCGGTATTTTTCG	
P38	CCGCTCGAGTTAAACTTCGTTGAATAAATAATTCC	
**Primer extension**		
E1	CCACAACCCCCCTAAAAACG	Figures S1, S6, 3, 6
E2	CCCTTAAAAGAAACACCTTAA	Figure 6
E3	CCGCTTTCAATTCAAGTGAATG	Figure S6

**HU cloning**		
H1	CACCGGATCCATGAACAAGCGGAATTTATTGATTTG	Figure S3, Table S1
H2	GGTAGTCGACTCACTTACCTTCTTCAACTTTTTGTT	

SUPPLEMENTARY METHODS

$KMnO_4$ footprinting

DNA modification was performed as described previously [38]. Mixtures containing 300 ng of plasmid DNA (approximately 10 nM), 25 mM Hepes-KOH (pH 7.6), 12% (v/v) glycerol, 1 mM $CaCl_2$, 0.2 mM EDTA, 5 mM ATP, 0.1 mg/ml BSA, and DnaA protein (up to 800 nM) in a total volume of 15 μL were incubated at 30°C. Subsequently, 3 μL of 48 mM $KMnO_4$ was added, and the incubation was continued for 5 min. The reaction was quenched by adding 1.5 μL of 2-mercaptoethanol and 1.5 μL of 0.5 M EDTA. The DNA was purified using GeneJET Purification columns and used as a template in PE reactions.

HU cloning, expression, and purification

The *H. pylori hu* gene (HP0835, 285 bp) was amplified by PCR using the H1–H2 primer pair and inserted between the BamHI-SaII restriction sites of pET151Nterm (Table S1) to generate pET151-HU. BL21 cells (1 l) harbouring the pET151-HU expression vector were grown at 37 °C until the optical density (OD_{600}) reached 1.0. Then protein synthesis was induced with 0.1 mM IPTG for 3 hours. The cultures were harvested by centrifugation (10 min, 5000*g*, 4°C). The cells were suspended in 50 mL of ice-cold sonication buffer HisA (50 mM NaH_2PO_4, 300 mM NaCl, 1 mM imidazole, pH 8.0 supplemented with SIGMAFAST™ Protease Inhibitor Cocktail Tablets, EDTA-Free), disrupted by sonication, and centrifuged (30 min, 26915×g, 4°C). The supernatant was mixed with a Ni-NTA His-Select Nickel Affinity Gel (0.25 mL bed volume, Sigma P6611). The proteins were incubated with the resin for 1 h on ice under gentle continuous agitation. To remove non-specifically bound proteins, the agarose beads were washed twice with 50 mL HisA buffer with 10 mM imidazole and once with 50 mL of HisA supplemented with 50 mM imidazole (after the washing steps, the Bradford test gave a negative result). The slurry with bound histidine-tagged proteins was transferred to the column and the elution was carried out with HisA buffer supplemented with 100 mM imidazole.

Electrophoretic Mobility Shift Assay (EMSA)

EMSA was conducted as described previously [37,40], using a FAM-labelled *oriC1* probe and 6HisHU protein. FAM-labelled DNA was incubated with DnaA protein at 30 °C for 20 min in a Marians' binding buffer (20 mM Hepes/KOH, pH 7.6, 5 mM magnesium acetate, 1 mM EDTA, 4 mM DTT (dithiothreitol), 0.2% Triton X-100, 5 mM ATP, and 100 ng mL^{-1} BSA). The bound complexes were separated by electrophoresis in a 4% polyacrylamide gel in 0.25× TBE (89 mM Tris/89 mM borate/1 mM EDTA) at 10 V/cm, 4°C. The gels were analyzed by a Typhoon 9500 FLA Variable Mode Imager and ImageQuant software.

Electron Microscopy (EM)

Electron microscopy was performed as described previously [37,38,40] with minor modifications: 90 ng of DnaA protein (approximately 110 nM) was incubated with 60 ng (approximately 1.4 nM) of plasmid DNA. The localization of the complex was measured using ImageJ software.

Mutagenesis

Pori Plasmids

To examine the role of *H. pylori* DUE and DUE-proximal modules in DNA unwinding and DnaA protein binding, a series of plasmids with mutagenized DnaA-trio motif (poriTrio1, poriTrio2, poriTrio4), GC-rich motif (poriG1-4), AT-rich region (poriAT1), and DnaA box ts1 (pori_ts1mut)

sequences were prepared. The plasmids (Table S1) were constructed in a two-step approach. The first nine pairs of PCR products were amplified using the poriWT plasmid as a template and specific primer pairs (Tables S1 and S2). Next, the PCR products were used as templates in an overlap extension PCR with primers P1 and P2 to obtain DNA fragments containing the designed mutations. The desired fragments were digested with EcoRI/PstI restriction enzymes, gel purified, and cloned into the poriWT plasmid digested with the same enzymes. The plasmid poriTrio3 required a three-step approach to change the polarity of the hs region and the DUE. The first four PCR reactions were performed using pairs of primers: P1/P17 and P2/P18 with poriWT as a template and P15/P20 and P16/P19, which were templates for each other. The PCR products were then used in a fusion PCR with primers P15 and P16. Finally, all three fragments were used as a template in PCR with oligonucleotides P1 and P2, and the product was cloned like that described above.

H. pylori *ts1mut construction*

The pori_ts1mut-*aphA-3* plasmid for the allelic exchange of the wild-type ts1 for the ts1mut DnaA box in the *H. pylori* chromosome was prepared by the overlap extension PCR method. The *aphA-3* region was amplified by P33–P35 primer pair using 26695Δ1021::*aph-3* genomic DNA as a template [49]. The 5′ fragment of *comH (HP1527)* was amplified by P34–P36 primer pair using *H. pylori* 26695 genomic DNA as a template. The PCR products were used as templates to synthesize the final PCR product *aphA-3-comH* amplified by P33- P35 primer pair. The *aphA-3-comH* PCR product was digested with PstI and cloned into pori_ts1mut digested with PstI and EcoRV giving the pori_ts1mut-aphA-3 vector. The *aphA-3-comH* PCR product was also cloned into the poriWT giving a pori_ts1WT-*aphA-3* vector, in which the ts1 box was as in the wild-type strain. *H. pylori* 26695 was transformed with the pori_ts1mut-*aphA-3* and pori_ts1WT-*aphA-3* plasmids and plated on BA plates supplemented with kanamycin. The *H. pylori* kanamycin-resistant mutants were then isolated and analyzed by PCR using E3-P38 to confirm that the *aphA-3* cassette was introduced. The mutation of the ts1 box was confirmed by sequencing. As a result, *H. pylori* 26695 ts1mut and ts1WT mutant strains were obtained.

SUPPLEMETARY FIGURES

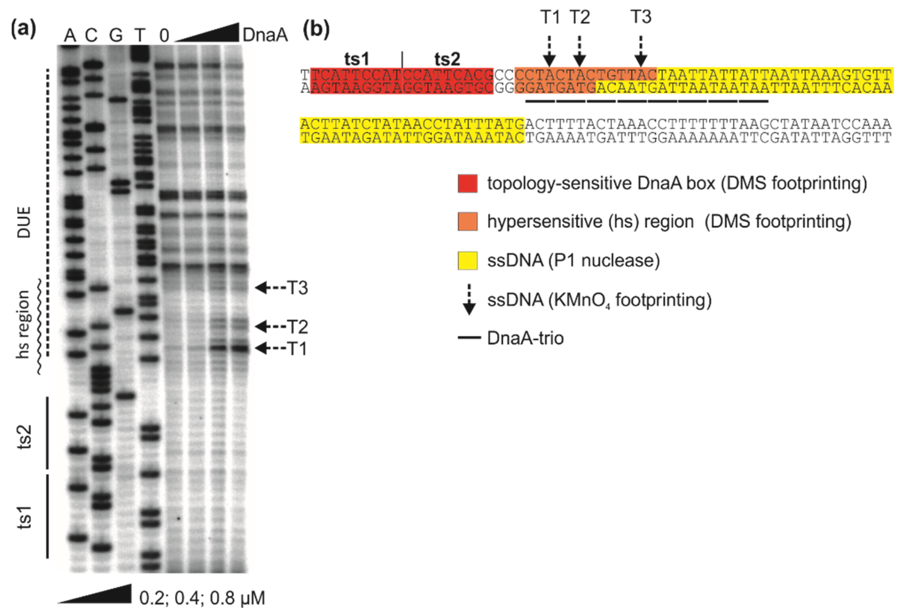

Figure S1. Precise mapping of the *H. pylori* 5′ DUE border. (**a**) $KMnO_4$ footprinting. $KMnO_4$ preferentially modifies pyrimidine bases, specifically thymine residues, located within single-stranded DNA. Thus, $KMnO_4$ footprinting permits monitoring of DNA helix status (ds or ss DNA). During primer extension (PE) reactions subsequent to $KMnO_4$ treatment, the polymerase halts at modified thymines; this yields additional bands on a denaturing gel compared to DNA not treated with $KMnO_4$. The poriWT plasmid was incubated with the indicated amounts of DnaA, treated with KMnO4, and used as a template in PE reactions with ^{32}P-labeled primer E1. The hs region, the DUE, and the ts boxes are indicated by wavy, dashed, and continuous lines, respectively. (**b**) Schematic representation of the DUE-proximal region in *H. pylori* (based on [37,40] and part (a) of this Figure).

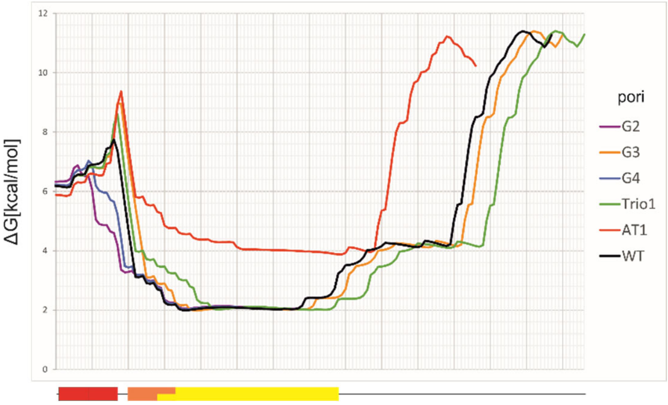

Figure S2. In silico analysis of stress-induced duplex destabilization (SIDD) in plasmids with introduced mutations. Mutations that differ in the destabilization profile from the wild-type sequence (WT) are depicted. A schematic representation of the analyzed sequence (WT) is provided beneath the plot; the most important features are marked as in Figure 1**.**

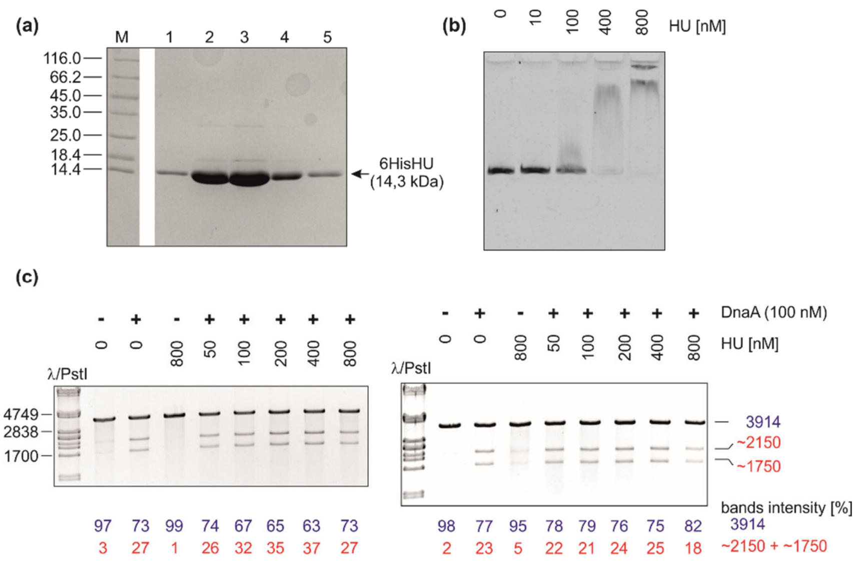

Figure S3. HU influence on DnaA-dependent *oriC* unwinding. (**a**) Quality of the purified recombinant 6HisHU protein; 10 µL of each eluted fraction was resolved in a 15% SDS-PAGE gel and stained with Coomassie Brilliant Blue. M, Pierce unstained protein MW marker; 1-5, eluted protein fractions. (**b**) Gel shift analysis of the interactions of *H. pylori* HU with DNA. FAM-labelled *H. pylori oriC1* (180 bp) was incubated with 6HisHU. The complexes were resolved in a 4% polyacrylamide gel. (**c**) DnaA -dependent DUE unwinding. The poriWT plasmid was incubated with the indicated amounts of DnaA protein and 6HisHU proteins, digested by P1 nuclease, purified, and digested by BglII (P1/BglII). The DNA fragments were resolved on 1% agarose gels and stained with ethidium bromide. The sizes of the λ/PstI DNA ladder bands and the expected DNA restriction fragments (in base pairs) are indicated to the left and right of the gel images, respectively. Digital processing was applied equally across the entire images, including controls. The results of two independent gel-shift analyses were analyzed densitometrically using ImageLab (BioRad). Each DNA band was quantified and is presented as a percentage of the intensity of total plasmid resolved in its lane.

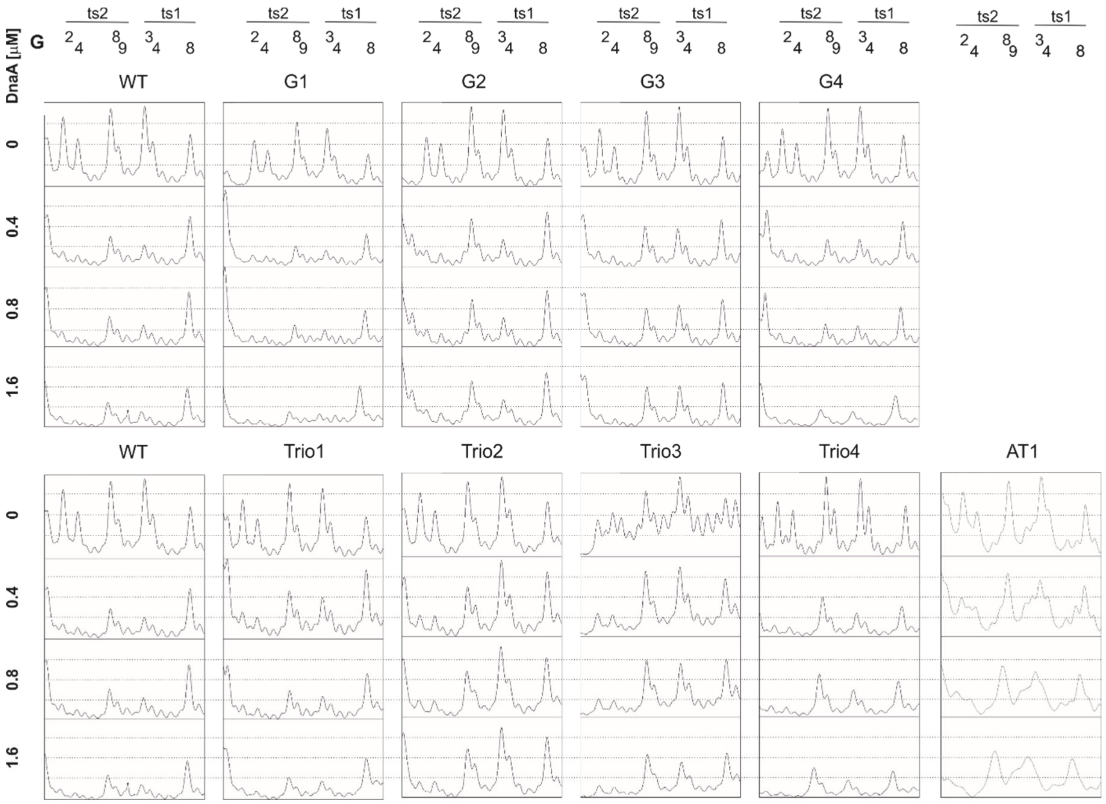

Figure S4. Densitometric analysis supplementing DMS analysis of DUE submodules mutations (Figure 3). Indicated concentrations next to the plots correspond to the analyzed lanes. Protected guanosine residues (G) with the position of protection for each DnaA box are indicated above the plots. The plot for the poriWT is repeated twice for a better comparison of plots presenting DMS results obtained with mutated pori plasmids.

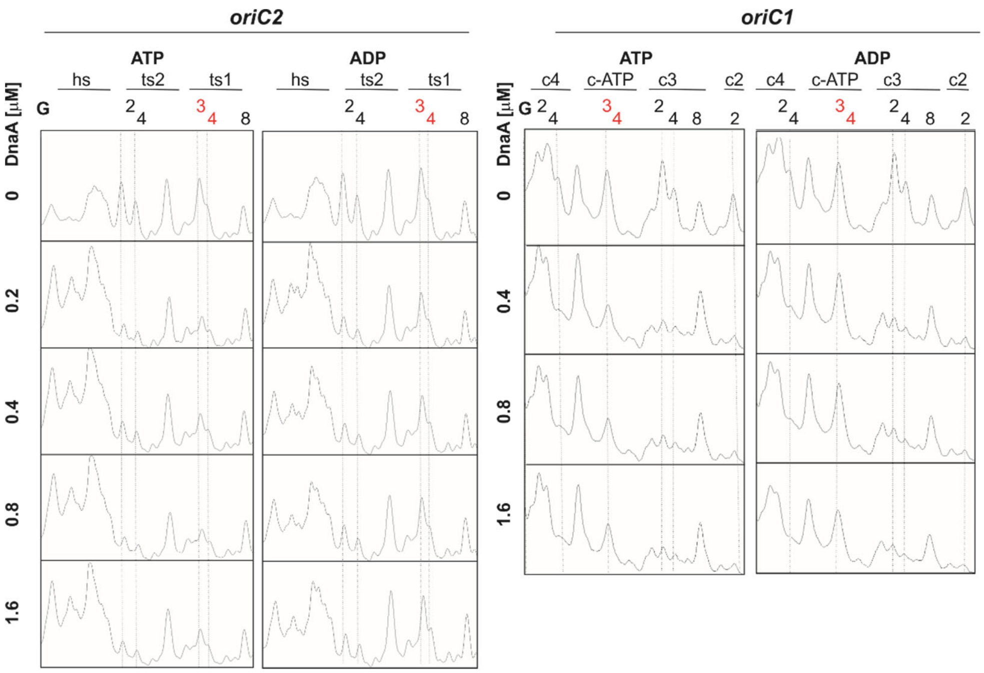

Figure S5. Densitometric analysis supplementing the identification of the ATP-dependent DnaA boxes (Figure 6). The concentrations indicated next to the plots correspond to the analyzed lanes. The protected guanosine residues (G) and the protected positions within each DnaA box are indicated above the plots and dotted lines.

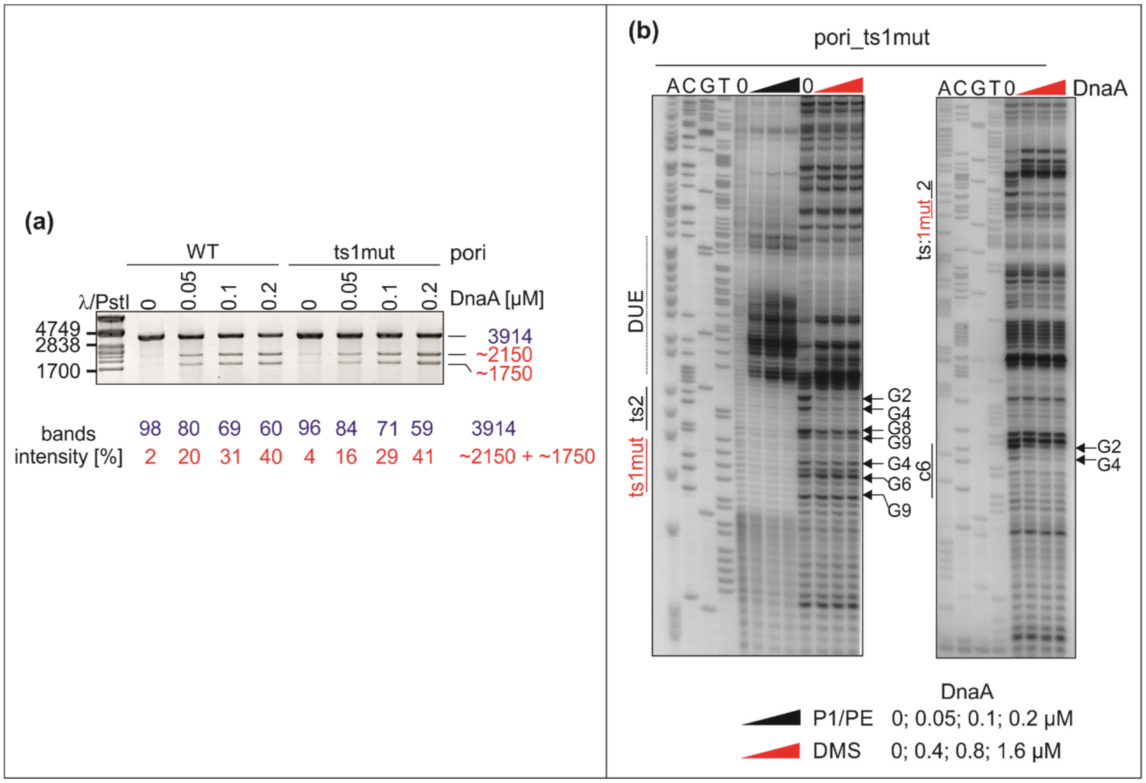

Figure S6. Analysis of the influence of the ts1 box mutation on the functionality of the *oriC* region. (**a**) DNA unwinding of poriWT and pori_ts1mut plasmids. The plasmids were incubated with the indicated amounts of DnaA protein, digested by P1 nuclease and digested by BglII (P1/BglII). The DNA fragments were resolved on 1% agarose gels and stained with ethidium bromide. The sizes of the λ/PstI DNA ladder bands and the expected DNA restriction fragments (in base pairs) are indicated to the left and right of the gel images, respectively. Each DNA bands was quantified and is presented as a percentage of the intensity of total plasmid resolved in its lane. (**b**) Analysis of DnaA binding to the ts1mut DnaA box and DUE unwinding. After incubation with the indicated amounts of DnaA, plasmid were treated with P1 nuclease or DMS and used as templates in PE reactions. The DUE and ts boxes are marked to the left of the gels, while DMS-modified residues in ts1-ts2 DnaA boxes (G) are labelled to the right of the gels. The mutation of box ts1 probably affected the interaction between DNA and DnaA because we were unable to detect a decrease in intensity of the cytosine band in the 6th position (G4) of the mutated DnaA ts1mut box sequence. The binding to G8 and G9 of the ts2 box was also reduced, but the interaction with G2 and G4 was efficient as in the poriWT; the interactions with c6 DnaA box and DNA looping remained intact (Figure S10). The previously identified DMS hypersensitive region was also detectable in the mutated plasmid. The region of increased methylation frequency in pori_ts1mut encompasses 15 nt, similarly to the wild-type sequence.

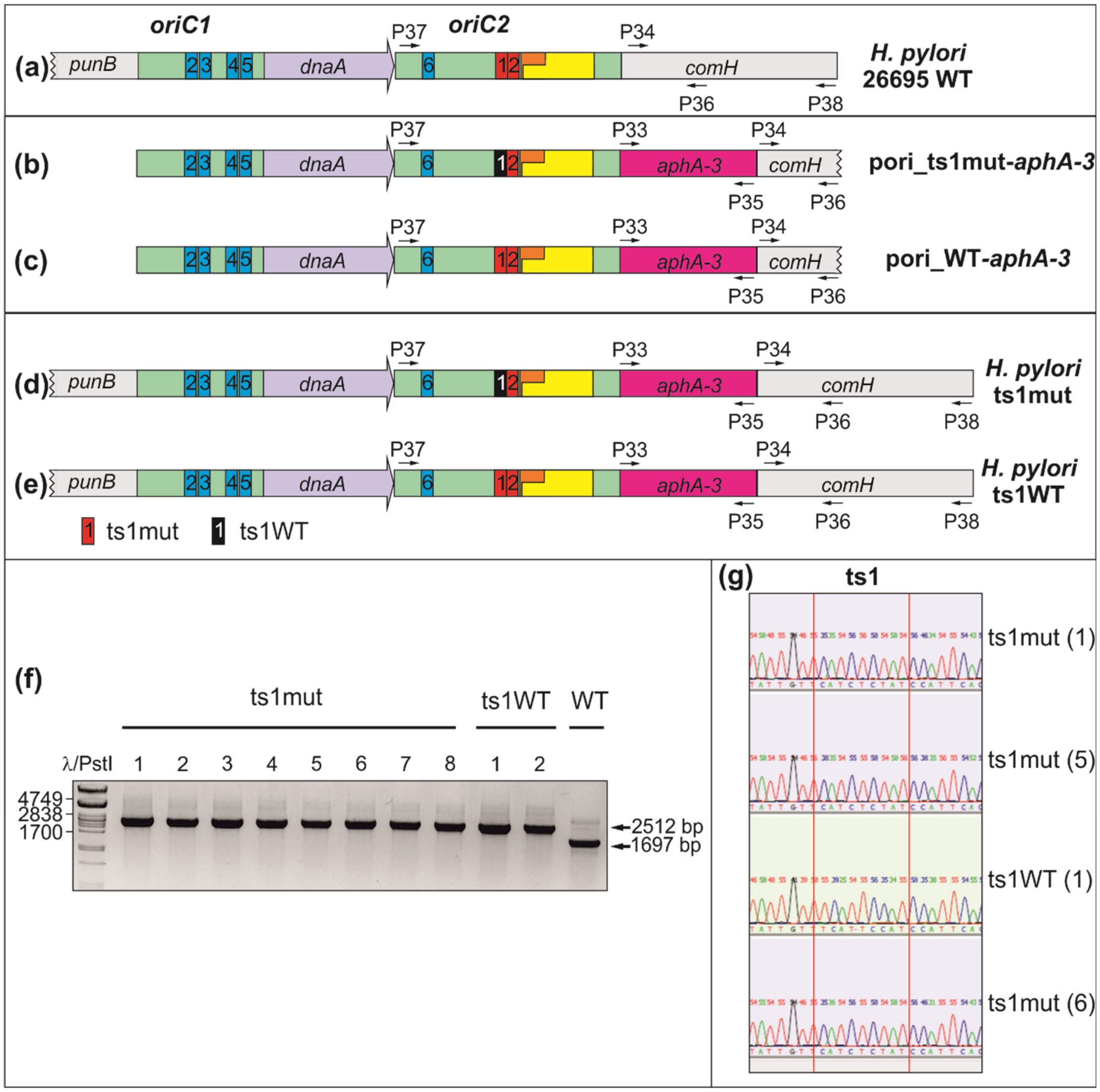

Figure S7. The mutagenesis strategy used to mutate the ts1 DnaA box on the *H. pylori* chromosome. *H. pylori* 26695 wild-type *oriC* chromosomal locus (**A**) and DNA fragments, which recombined with the *H. pylori* chromosome via double crossing-over to give *H. pylori* ts1mut (**B, D**) and ts1WT (**C, E**) mutant strains, are shown. Primer sequences are given in Supplementary Table *S2*. (**F**) PCR analysis of genomic DNA isolated from the *H. pylori* wild-type and mutant strains. Eight individual kanamycin-resistant ts1mut and two ts1WT colonies were analyzed using E3-P38 primer pair (Table S2). (**G**) Analysis of ts1mut mutation in three clones of *H. pylori* ts1mut and one clone of tsWT mutant strains. *oriC2* was amplified by the E3–P37 primer pair and sequenced by Sanger sequencing. The ts1 DnaA sequence is boxed.

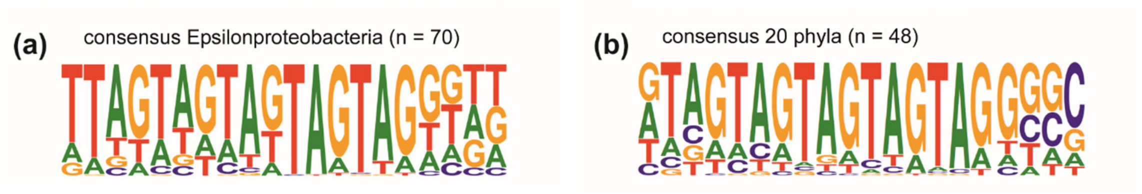

Figure S8. DnaA-trio motifs in the *oriC* replication origins of Epsilonproteobacteria (**a**) and bacteria from more than 20 different phyla (**b**). Complete bacterial genome sequences were obtained from GenBank except for the known *oriCs* of *Bacillus subtilis* (NCBI:txid224308), *Bdellovibrio bacteriovorus* HD100 (NCBI:txid264462), *Synechococcus elongatus* PCC 7942 (NCBI:txid1140), *Thermatoga maritima* MSB8 (NCBI:txid243274), and *Thermus thermophilus* HB8 (NCBI:txid300852). Origin predictions were performed as described previously [42].

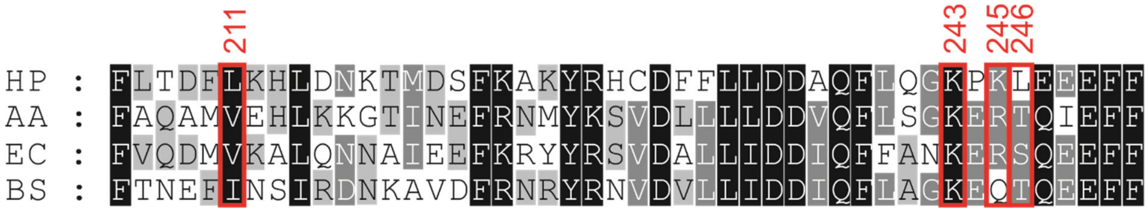

Figure S9. Alignment of the amino acid sequences of DnaA of *H. pylori* (HP), *A. aeolicus* (AA), *E. coli* (EC), and *B. subtilis* (BS). Red rectangles mark residues located within the ISM and B-motifs that are important for the interaction of *E. coli* DnaA with ssDNA. The red numbers indicate the positions of amino acids in *E. coli* DnaA.

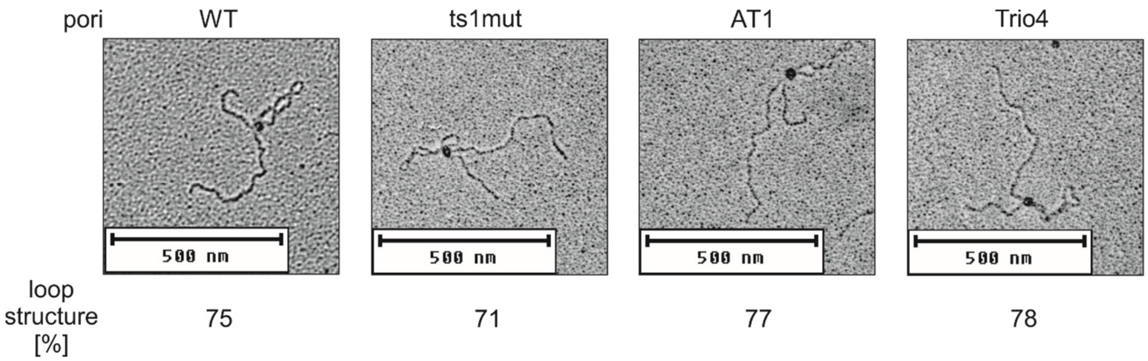

Figure S10. Representative electron micrographs of selected mutated plasmids presenting structures formed by DnaA protein interactions with suborigins. The frequency of loop structure formation was calculated based on the analysis of 300 molecules bound by DnaA for each plasmid.
